# Temperature and E-Poling Evolution of Structural, Vibrational, Dielectric, and Ferroelectric Properties of Ba_1−x_Sr_x_TiO_3_ Ceramics (x = 0, 0.1, 0.2, 0.3, 0.4 and 0.45)

**DOI:** 10.3390/ma16186316

**Published:** 2023-09-20

**Authors:** Jan Suchanicz, Dorota Sitko, Krzysztof Stanuch, Konrad Świerczek, Grzegorz Jagło, Andrzej Kruk, Kamila Kluczewska-Chmielarz, Krzysztof Konieczny, Piotr Czaja, Jakub Aleksandrowicz, Wojciech Wieczorek, Justyna Grygierek, Mariusz Sokolowski, Grzegorz Stachowski, Maija Antonova, Andris Sternberg

**Affiliations:** 1 Department of Mechanical Engineering and Agrophysics, University of Agriculture in Krakow, Balicka 120, 31-120 Krakow, Poland; 2 Faculty of Exact & Natural Sciences, Pedagogical University, ul. Podchorazych 2, 30-084 Krakow, Poland; 3 Institute of Physics, Pedagogical University, ul. Podchorazych 2, 30-084 Krakow, Poland; 4 Department of Hydrogen Energy, Faculty of Energy and Fuels, AGH-University of Science & Technology, al.Mickiewicza 30, 30-059 Krakow, Poland; 5 Institute of Technology, Pedagogical University, ul. Podchorazych 2, 30-084 Krakow, Poland; 6 Faculty of Materials Science and Ceramics, AGH-University of Science & Technology, al.Mickiewicza 30, 30-059 Krakow, Poland; 7 Faculty of Computer Science, Electronics and Telecommunications, AGH-University of Science & Technology, al.Mickiewicza 30, 30-059 Krakow, Poland; 8 Astronomical Observatory, Jagiellonian University, Orla 171, 30-244 Krakow, Poland; 9 Institute of Solid State Physics, University of Latvia, LV1067 Riga, Latvia

**Keywords:** barium-strontium titanate BST, ab initio calculations, electronic band structure, structural properties, dielectric properties, optical properties, ferroelectric properties

## Abstract

Lead-free Ba_1−x_Sr_x_TiO_3_ (BST) (x = 0, 0.1, 0.2, 0.3, 0.4 and 0.45) ceramics were successfully prepared via the solid-state reaction route. A pure perovskite crystalline structure was identified for all compositions by X-ray diffraction analysis. The basic phase transition temperatures in these ceramics were studied over a wide temperature range. A change in symmetry from a tetragonal to cubic phase was detected, which was further proven by phonon anomalies in composition/temperature-dependent Raman spectra. The incorporation of Sr^2+^ into BaTiO_3_ (BT) lead to a shift in the phase transitions to lower temperatures, suppressing the ferroelectric properties and inducing relaxor-like behavior. Therefore, it was reasonable to suppose that the materials progressively lack long-range ordering. The initial second-harmonic generation (SHG) measurements demonstrated that the cubic phase of BST ceramics is not purely centrosymmetric over a wide temperature interval. We discussed the possible origin of the observed effects, and showed that electric field poling seems to reconstruct the structural ordering destroyed by the introduction of Sr^2+^ to BT. In the first approximation, substitution of Sr for larger Ba simply reduced the space for the off-central shift in Ti in the lattice and hence the domain polarization. A-site cation ordering in BST and its influence on the density of electronic states were also explored. The effect of doping with strontium ions in the BST compound on the density of electronic states was investigated using ab initio methods. As the calculations showed, doping BT with Sr^2+^ atoms led to an increase in the bandgap. The proposed calculations will also be used in the subsequent search for materials optimal for applications in photovoltaics.

## 1. Introduction

Titanate-based perovskites like BaTiO_3_, SrTiO_3_, (Ba,Sr)TiO_3_ are an important family of materials widely used as ceramic capacitors, microwave phase shifters and thermoelectric materials [1,2,3,4,5,6,7,8,9,10]. La-doped SrTiO_3_ was developed as a solid oxide fuel cell anode material [11]. Highly conductive Nb-doped SrTiO_3_ wafers are widely used as thin film substrates.

Barium titanate BaTiO_3_ (BT) undergoes three successive first-order phase transitions: from cubic (Pm3m) to tetragonal (P4mm) phase at ~120 °C, then to an orthorhombic (Amm2) phase at ~5 °C, and finally to a rhombohedral (R3m) phase at ~−90 °C. The cubic phase is paraelectric, whereas the other phases are ferroelectric. BT is commonly regarded as a displacive-type ferroelectric material, although some theoretical and experimental results point to an order–disorder component during phase transition [12,13,14,15,16]. It has been postulated that the fluctuating order parameter originates from the presence of electric dipoles, induced by hopping of the Ti ion around its equilibrium position at temperatures far above T_c_, similar to relaxor materials [17]. However, the role and nature of displacive and order–disorder components during phase transformation is still not fully understood.

SrTiO_3_ (ST) is a typical perovskite compound possessing cubic symmetry at room temperature. Below −168 °C, a transition to tetragonal symmetry was reported [18,19]. The relatively high electric permittivity combined with the low dielectric loss of ST can be interesting for various microwave applications. It was reported that this material is an incipient ferroelectric whose ferroelectricity is suppressed by quantum fluctuations [20,21].

As pure BT and ST demonstrate different properties, the behavior of Ba_1−x_Sr_x_TiO_3_ (BST) solid solution is expected to evolve with composition. BST ceramics have been investigated since the middle of the last century; however, this interesting system has not been thoroughly investigated in recent years. Much of the research was concerned with the composition-related influence on T_c_ and the dielectric properties of BST [22,23,24]. More attention was paid to understanding the defect chemistry of both pure and acceptor/donor-doped Ba_1−x_Sr_x_TiO_3_ [2,3,4,5,6,25,26,27,28,29]. There are a number of published reports on the vibrational and ferroelectric properties of Ba_1−x_Sr_x_TiO_3_. Raman and Fourier transform infrared spectroscopy (FTIR) studies were presented in the literature [29,30,31,32,33]. Most of the reports did not deliberate on the effect of electric poling. The use of ab initio methods to calculate the properties of BST structures allows for the theoretical values of physical properties to be determined in order to both compare them with experimental data for these compounds, and to predict their values in its absence. In addition, there are few theoretical works on the studied structure (tetragonal/ferroelectric). However, the electronic structure (valence states of ions) plays an essential role in electric (ferroelectric) ordering.

Wet chemical methods, including hydrothermal synthesis, co-precipitation and sol–gel, allows for the production of homogeneous, ultrafine, and high-purity powders. Nevertheless, these methods are time-consuming and require commercially costly chemicals. Solid-state reaction is the most accurate at analyzing materials for commercial purposes. The grain size and surface area of powders can be controlled by the milling process, leading to the production of fine powders.

Based on these considerations, we undertook the synthesis of Ba_1−x_Sr_x_TiO_3_ ceramics for x = 0, 0.1, 0.2, 0.3, 0.4 and 0.45 (BT, BST1, BST2, BST3, BST4 and BST4.5) to produce and thoroughly investigate their structural, vibrational, dielectric, and ferroelectric properties in order to obtain a better insight into various aspects of their behavior. The studies were complemented with the investigation of the second harmonic generation (SHG) signal and FTIR spectroscopy. Although many properties of BST have been presented in the literature, they have not provided such a comprehensive picture of the system. It should be noted that complementary studies performed on specimens prepared with the same procedure make it possible to present a more general view of this interesting system.

## 2. Materials and Methods

The conventional solid-state sintering process was used to prepare the Ba_1−x_Sr_x_TiO_3_ ceramics (x = 0 (BT), 0.1 (BST1), 0.2 (BST2), 0.3 (BST3), 0.4 (BST4) and 0.45 (BST4.5)). Reagent-grade powders of BaCO_3_ (99.5%, Sigma Aldrich, St. Louis, MO, USA), TiO_2_ (99.9%, POCH) and SrCO_3_ (99.5%, Sigma-Aldrich) were used as raw materials, which were weighed according to the corresponding formula and then ball-milled in ethanol for 24 h with 600 rpm·min^−1^ For this purpose, a pure zirconium ball mill was used together with zirconia balls. Firstly, the calcination was performed at 1300 °C for 2 h with heating and cooling rates of 2 °C min^−1^. Secondly, these calcined powders were compacted into green disks at a pressure of 100 MPa. Sintering was carried out at 1430 (for BT)-1450 °C (for BST4.5) for 1.5 h in air. The heating and cooling rates were kept at 2 °C min^−1^. The relative density of the samples immersed in a water as measured by Archimedes’ method was 97%.

The crystalline structure measurements were performed by the X-ray diffraction (XRD) method (using a Panalytical Empyrean Diffractometer with CuK_α_). The XRD patterns were collected in the 2θ range of 65–85 degrees with step size of 0.05 (2θ). Rietveld analysis of the XRD data was supported by GSAS/EXPGUI software II. The high temperature HT-XRD measurement were conducted from −230 to 300 °C at selected temperatures.

The microstructure analysis was undertaken by an electron scanning microscope (SEM, Model Hitachi S−4700) with field emission and the Noran Vantage system. SEM images were obtained by applying accelerating voltage of 10 kV. Measurements of linear grain size and distribution were performed by means of the ImageJ 1.6 software, using a series of binarized SEM images. The surfaces of the samples were chemically treated with 5 vol% HCl + 0.5 vol% HF + 94.5 vol% H_2_O solution. Samples were kept in this solution for 7 s and then washed with distilled water.

Dielectric measurements were carried out on the plate-shaped samples with silver electrodes using a GW 821 LCR meter in a temperature/frequency ranging from −130 °C to 300 °C and from 100 Hz to 2 MHz, respectively. The electrodes were connected to the sinters using silver paste. The data were collected regularly with a step of 0.1 °C on heating and cooling, at a rate of 1.5 °C min^−1^, using an automatic temperature controller.

Raman measurements were carried out using a Horiba LabRam HR Raman Spectrometer equipped with 532 nm diode laser. The spectra were collected with a resolution of 1 cm^−1^. The experimental curves were performed using PeakFit (Systat) v.4.12 software package.

Differential scanning calorimetry (DSC) was performed by 200 F3 Maia Netzsch at temperatures ranging from −100 °C to 540 °C. Experiments were performed at a heating rate of 10 K-min^−1^ in an argon atmosphere at a flow rate of 50 mL min^−1^. The tested samples at weights of approximately 60 mg, were placed in a standard aluminum container.

The hysteresis loops were measured with the use of a Sawyer–Tower circuit at a frequency of 50 Hz. AN SDS 200A PC based Digital Storage Oscilloscope (Soft DSP) was used to record the hysteresis loops.

The pyroelectric current measurements of previously polarized samples were performed using a quasistatic method with heating at a rate of 10 °C min^−1^ using a Keithley electrometer (Model 6517A). The polarizing procedure proceeded from 150 °C down to room temperature under a DC electric field of 15 kVcm^−1^ using the High Voltage Power Supply (Spellman, Bertan, Series 230, Hauppauge, NY, USA). Remnant polarization was calculated by the integration pyroelectric current normalized by the sample area perpendicular to the current, and then plotted versus the corresponding temperature.

To obtain the poled state of the samples, the polarizing procedure was the same as that for the pyroelectric measurements.

A Q-switched laser, emitting radiation at λ = 1064 nm with a repetition rate of 1 kHz and a pulse width of 500 ps was used for the SHG measurements. The fundamental beam energy was controlled by rotating a half-wave plate and the residual fundamental light was removed by a 10 nm bandpass interference filter centered at 532 nm. The SHG intensity was detected by a photomultiplier and the SHG signal was controlled to eliminate any residual fluorescence. SHG measurements were performed on samples in unpoled and poled states with the same conditions as that for the pyroelectric current measurements.

FTIR studies were performed using a Bruker Vertex 70v vacuum spectrometer. A Harrick Scientific External Reflection attachment (Seagull) was employed. A total of 256 scans were recorded with a resolution of 4 cm^−1^.

**Figure 1:**
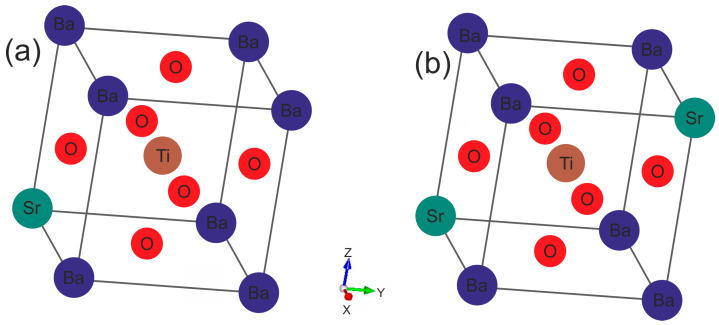
Crystalline structures of ferroelectric Ba_1−x_Sr_x_TiO_3_ perovskites at x = 0.125: (**a**) 0.25; and (**b**) with a tetragonal lattice.

Calculations were performed without the spin-polarized density functional theory, using the Vienna Ab Initio Simulating Package (VASP) [34,35]. Electron–ion interactions were represented by pseudopotentials as part of the projector-augmented wave (PAW) scheme. The valence electron configurations of the dataset were as follows: Ba: 5s^2^5p^6^6s^2^, Sr: 4s^2^4p^6^5s^2^, Ti: 3d^2^4s^2^, O: 2s^2^2p^4^. The exchange–correlation potentials were handled by applying the Perdew–Burke–Ernzerhof (PBE) [36] form of generalized gradient approximation (GGA). Such functionals are sufficiently accurate for the description of the electronic properties of perovskite systems [37,38]. For the Ba_0.875_Sr_0.125_TiO_3_ (Figure 1a) and Ba_0.75_Sr_0.25_TiO_3_ (Figure 1b) solid solutions, an ordered supercell of 2 × 2 × 2 with 40 atoms in the tetragonal cell was constructed. Computations were performed for one of the many possible Ba_1−x_Sr_x_TiO_3_ ion placements by substituting one and two Ba ions with one and two Sr ones. The sampling of Brillouin zones of 2 × 2 × 2 (40 atoms) Ba_1−x_Sr_x_TiO_3_ unit cells was performed using 8 × 8 × 7 Monkhorst–Pack k-point meshes. The tested structures were completely relaxed with a mesh of 8 × 8 × 7. While calculating the projected density of states, the k-space mesh was likewise 8 × 8 × 7. In the computations, a plane-wave basis with a cutoff energy ENCUT of 520 eV was used. The convergence criteria for the systems’ remaining forces and total energy were set to 10^−5^ eVÅ^−1^ and 10^−6^ eV.

## 3. Results and Discussion

**Figure 2:**
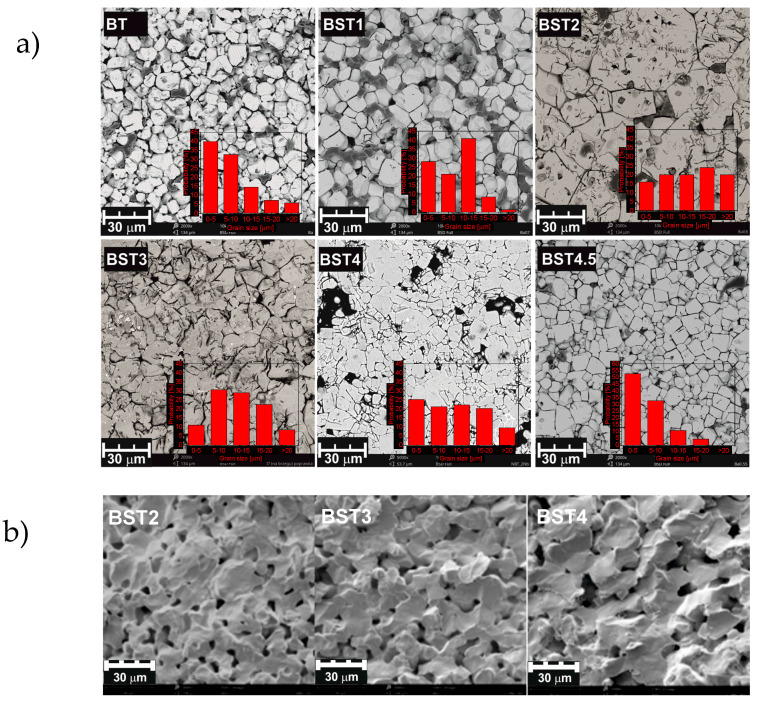
SEM micrographs of polished and chemically etched surface of BT, BST1, BST2, BST3, BST4, BST4.5 (**a**); and of the samples’ fractures of BST2, BST3 and BST4 (**b**) ceramics. Inset (**a**) the grain size distribution.

SEM (scanning electron microscope) micrographs of the polished and chemically etched surfaces of the investigated ceramics are shown in Figure 2a. In pure BT, the grain size reaches 9–19 μm. Grain growth is first hastened, and the grain distribution appears to be narrow. Second, it is inhibited and then again hastened upon the incorporation of strontium (see Supplementary Materials Figure S1). The grain size obtained from the BT, BST1 and BST4.5 sinters were composed of micro-size particles with a narrow size distribution ranging from 1 to 20 μm. The remaining samples were characterized by larger grain sizes with a diameter of up to several dozen μm. It should be highlighted that micro- size grains with sizes under 10 μm constituted the largest fraction in the BT sinter.

As can be seen, rather non-uniform grain shapes occur (except for pure BT). Some pores are also visible. Figure 2b shows SEM micrographs of the samples’ fractures. The microstructure images reveal a network of connected pores formed between the sintered grains. The grains are characterized by a smooth surface, but the addition of strontium weakens this effect. Notably, irregularities can be observed on the surface of BST4 grains, which likely stem from the grain growth. As can be seen, the surfaces of the fractures run on both the grain and the inter-grain boundaries. The SEM images show the formation of homogeneous and regular-shaped ceramic grains with clear visible boundaries.

**Figure 3:**
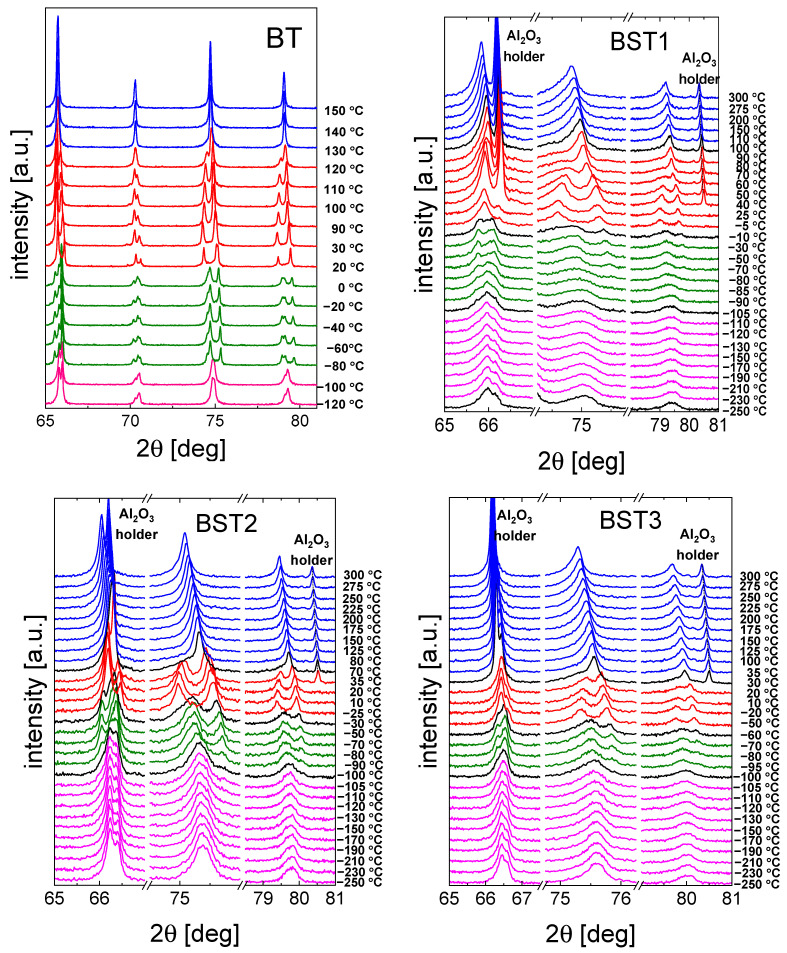

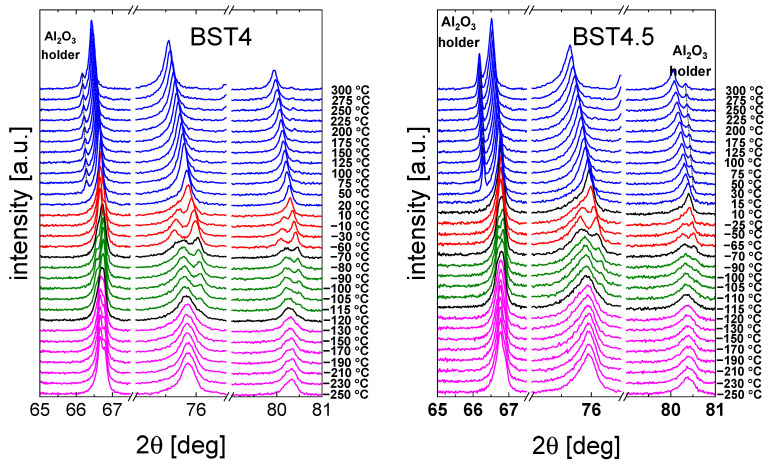
Selected range of XRD data of BT, BST1, BST2, BST3, BST4, BST4.5 ceramics. Colors indicate different crystal structures: blue—cubic, red—tetragonal, green—orthorhombic, and magenta—rhombohedral. In the case of a coexistence of two phases, X-ray diffraction patterns are presented in black.

A selected range of representative XRD diffractograms recorded for the ceramics is shown in Figure 3. The expected occurrence of the structural transformations comprising (from the lowest temperatures) rhombohedral ↔ orthorhombic ↔ tetragonal ↔ and cubic phases can be easily seen (see also Table 1). In particular, starting from the high-temperature cubic (paraelectric) phase, a transition to the tetragonal (ferroelectric) can be observed with splitting of the cubic peaks. This transition for the BST1 material starts to become visible in the data measured at about 110 °C; however, for BST4.5, the stability range of the cubic phase extends down to 15 °C, with the emergence of the tetragonal phase slightly visible at 10 °C. As can be seen from Figure 3, this particular phase transformation is indeed strongly affected by the doping level in the whole study series, in which increasing amounts of ST result in an extension of the cubic phase stability range. Simultaneously, temperatures of the remaining transformations are not as strongly affected by the chemical composition. The coexistence of particular phases (beginning of the transition) is indicated in black in the XRD spectra. For compounds with x ≥ 0.4, some broadening of the reflections of the cubic phase was observed. This may be the result of the temperature evolution of polar nanoregions existing in the cubic matrix (see below). Moreover, a decrease in lattice volume and tetragonal distortion was observed with an increasing amount of ST (Table 2). The appearance of the cubic phase for such materials can also be understood as a result of an increase in the structural disorder and the presence of a statistical mixture of different chemical bonds. The Ba–O bond is more ionic, but the Sr–O bond is mainly covalent. Therefore, the addition of Sr should increase the A-site disorder in the BT–ST system. This hampers the displacements of ferroelectric ions along the preferred direction (001)_c_, and the tetragonal distortions are suppressed [39]. The main effect of E-poling is sharper and more symmetric in the XRD peaks than in the unpoled ones, showing that the degree of order is enhanced under the electric field applied (not shown here).

**Figure 4:**
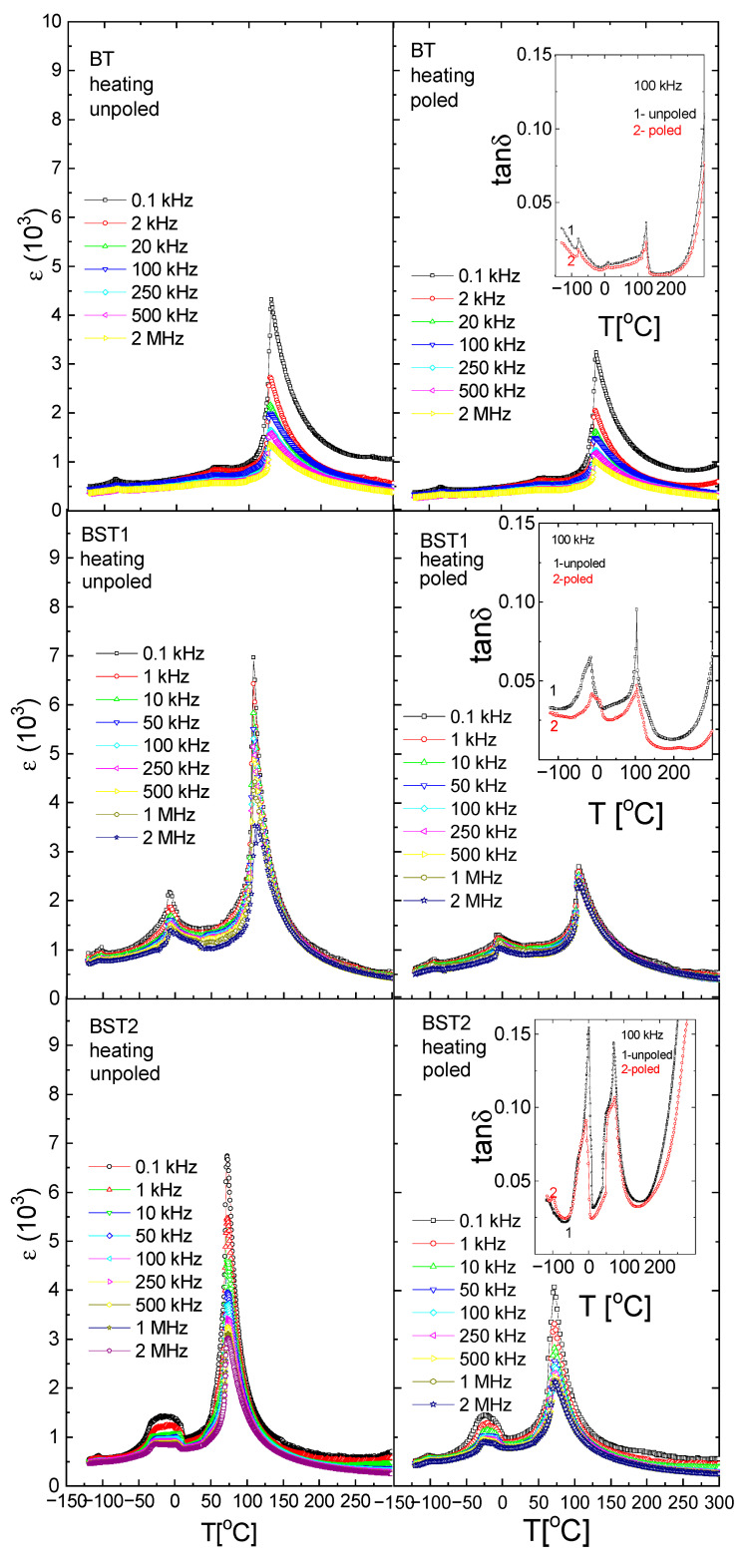

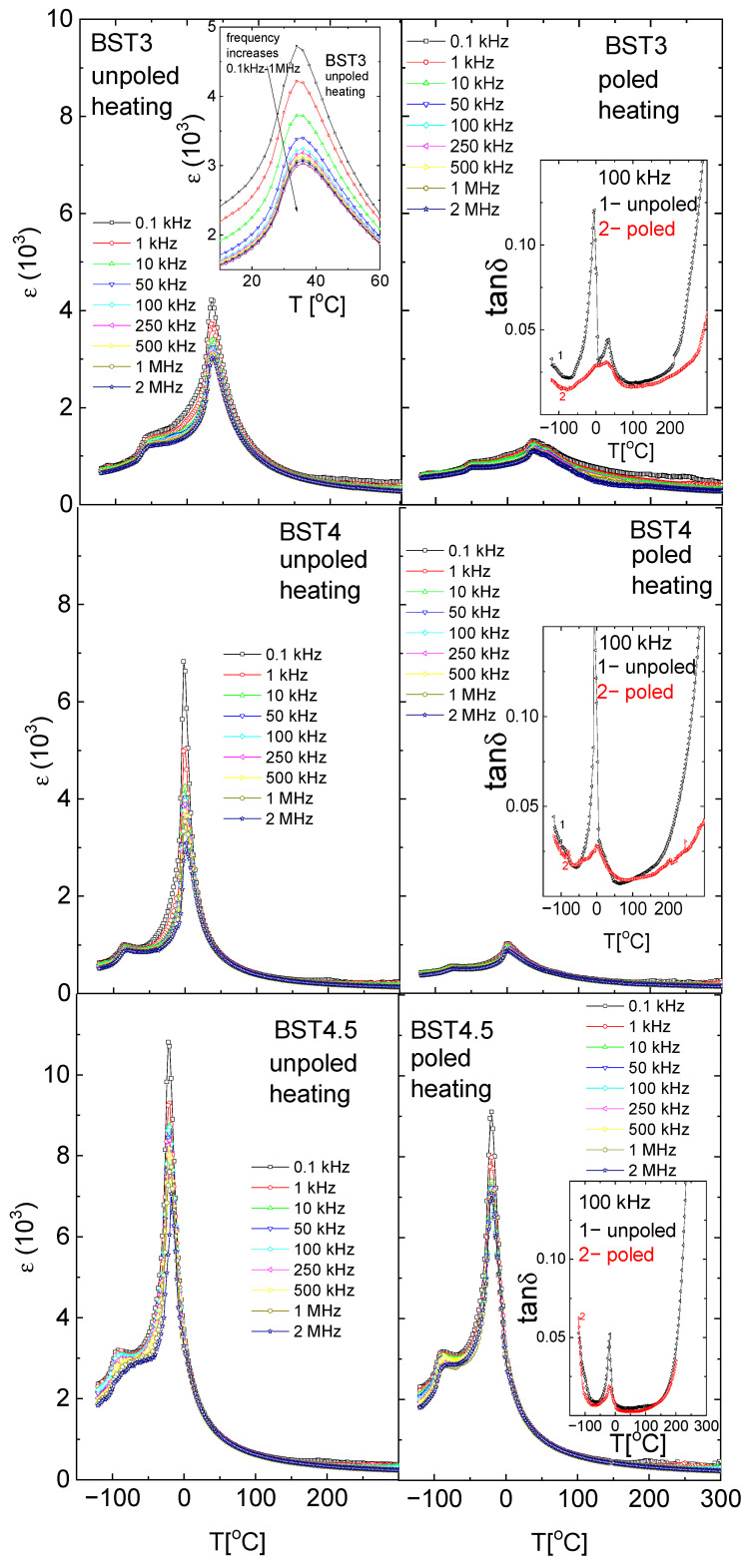
Temperature/frequency evolution of electric permittivity of BT, BST1, BST2, BST3, BST4 and BST4.5 ceramics. The insert on the **left** side of Figure 4 for BST3 shows the expanded part of the figure showing relaxor-like behavior. Inserts on the **right** side of Figure 4 show the temperature evolution on the dielectric losses tan δ at a frequency of 100 kHz.

Figure 4 shows the temperature/frequency dependence of the electric permittivity of Ba_1−x_Sr_x_TiO_3_ ceramics. The dielectric studies confirmed the presence of three phase transitions in the pure BT: rhombohedral-orthorhombic (T_O-T_) at −90 °C, orthorhombic-tetragonal (T_R-O_) at ~5 °C and tetragonal-cubic (T_O-T_) at ~120 °C, connected with a maximum of ε. These maxima shift towards a lower temperature with increasing amounts of ST. The ε(T) peaks are slightly broadened, dielectric dispersion increases with increasing amounts of ST, and the temperature corresponding to the permittivity maximum shifts slightly toward higher temperatures as the frequency increases (revealing relaxor-like behavior, insert on the left side of Figure 4 for BST3). The phase transition temperatures are summarized in Table 1. Similar to ε behavior, the maxima connected with the maximum of tan δ shift towards lower temperatures with increasing amounts of Sr (inserts on the right side of Figure 4). Both electric permittivity and dielectric losses decrease as a result of electric field action. This can occur as a result of improvement to the electric ordering by the electric field, previously disturbed by the substitution of Sr^2+^ ions into BT.

**Figure 5:**
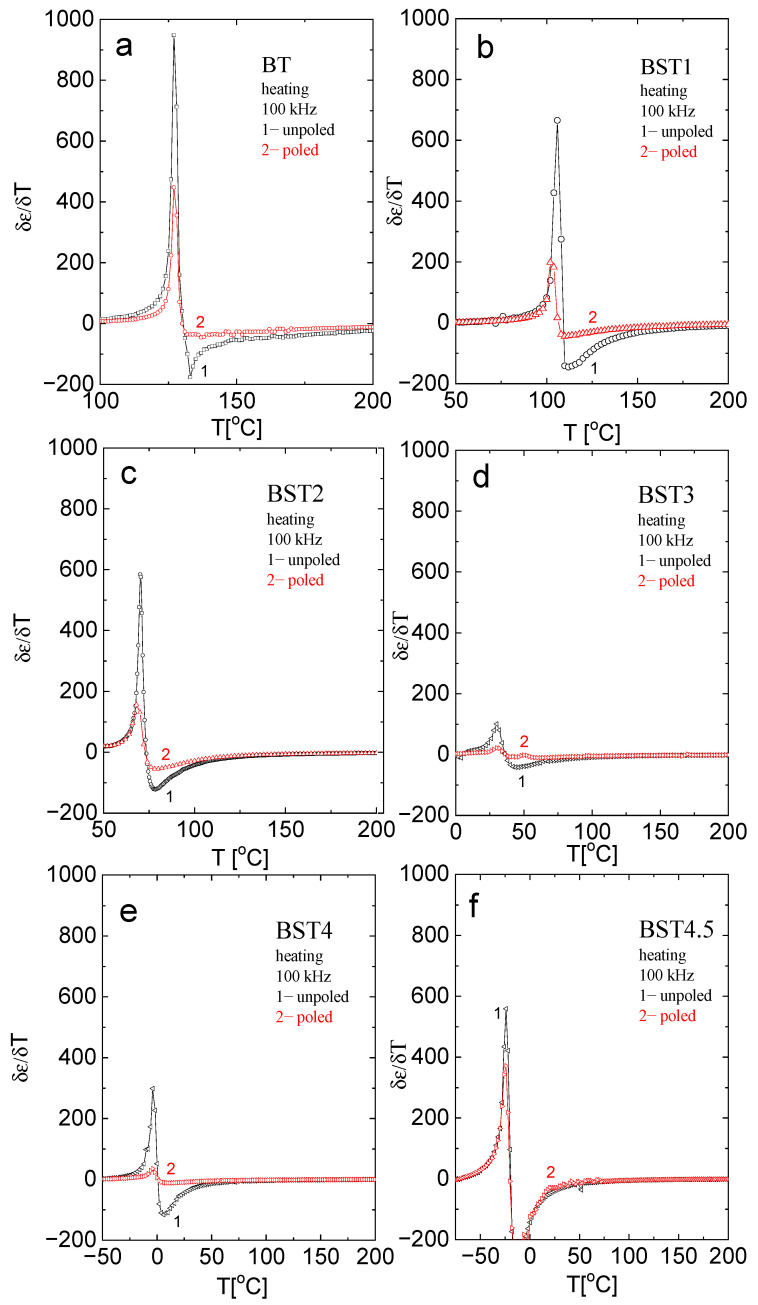
Temperature evolution of δε/δT near the tetragonal-cubic phase transition for: (**a**) BT; (**b**) BST1; (**c**) BST2; (**d**) BST3; (**e**) BST4; and (**f**) BST4.5 compositions.

For a better analysis of the character of the phase transition as a function of ST concentration, the (δε/δT) − T function was plotted in Figure 5. As can be seen, the sudden inflection in the function near T_m_ becomes more gradual as the ST concentration increases up to x = 0.3. As the sudden inflection is a signature of first-order behaviour, one can conclude that the behaviour tends to change to second-order. However, for x > 0.3, the sudden inflection seems to increase again, indicating a return to first-order behaviour.

The ε(T) curves for poled samples are similar to those for unpoled ones. However, electric permittivity and dielectric dispersion decrease in comparison to the unpoled samples; the maximum of electric permittivity connected with the phase transitions slightly shifts toward higher temperatures, and the sudden inflection in the (δε/δT)-T function for poled samples decreases. These indicate that E-poling tends to increase the degree of order previously disturbed by the incorporation of ST.

**Figure 6:**
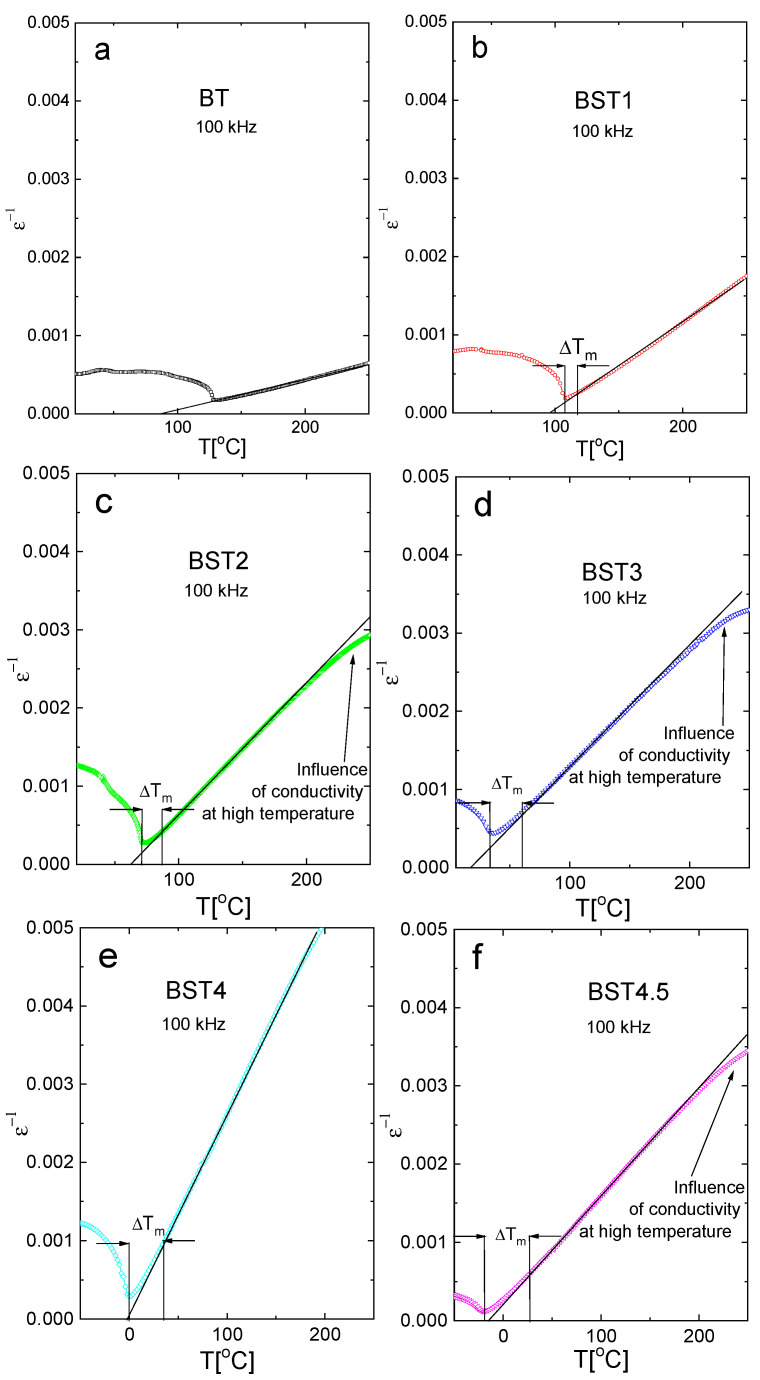
(ε^−1^)-T function of: (**a**) BT; (**b**) BST1; (**c**) BST2; (**d**) BST3; (**e**) BST4; and (**f**) BST4.5 ceramics.

It is well known that above T_c_, electric permittivity in ferroelectrics follows the Curie–Weiss law:

ε = C·(T − T_cw_)^−1^ (1)

where C is the Curie–Weiss constant and T_cw_ is the Curie–Weiss temperature. Figure 6 presents the (ε^−1^)-T function. As can be seen, the linear relationship between ε^−1^ and T is valid for undoped BT. However, this relationship is not valid for the remaining samples (strictly speaking, the relationship is not valid over some temperature intervals above ΔT_m_ = T_B_ − T_m_, where T_B_ is the Burns temperature; below which ε^−1^ does not linearly follow the temperature). ΔT_m_ is a measure of the extent of the deviation from the Curie–Weiss law. As is shown in Figure 6, ΔT_m_ increases with increases in ST content, indicating the increased diffuseness of the phase transition. In addition, the difference between the Curie–Weiss temperature T_cw_ and T_m_ decreases with increasing ST concentrations, which also suggests a trend towards second-order behavior.

**Figure 7:**
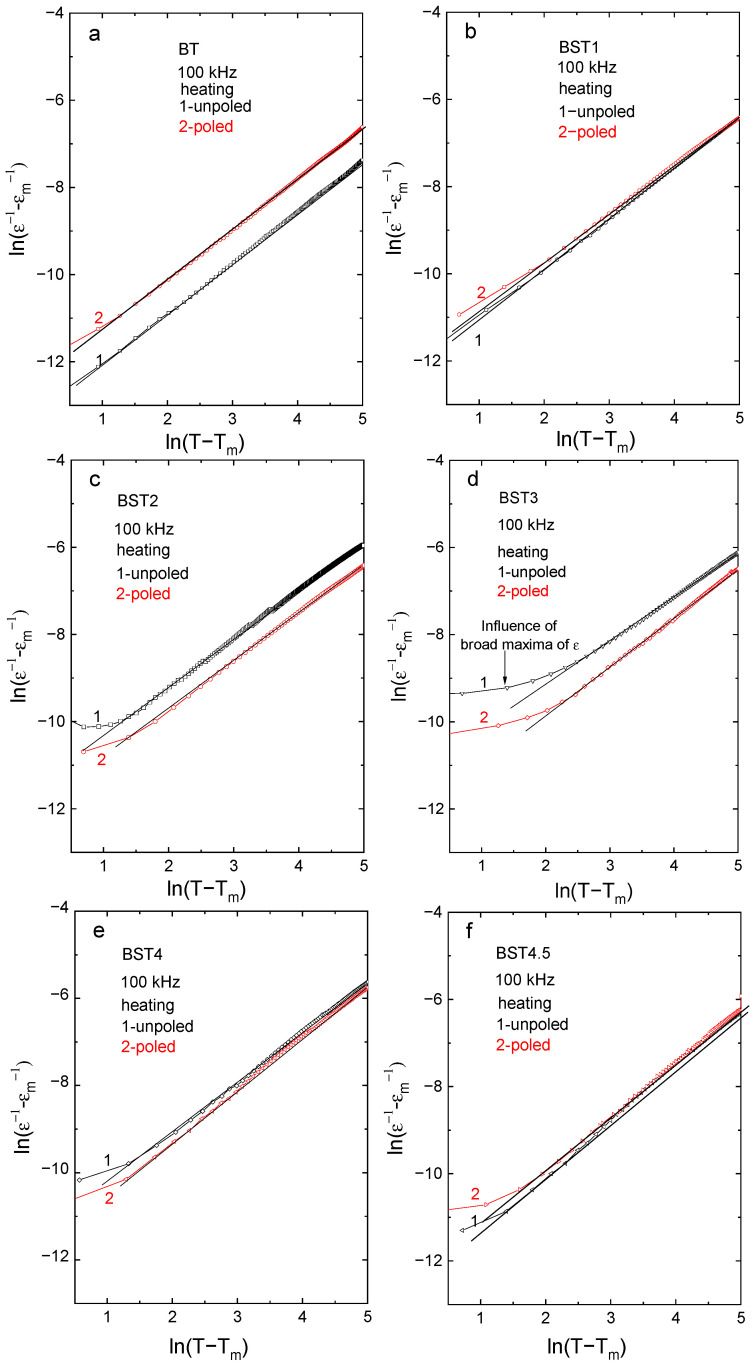
(ε^−1^ − ε_m_^−1^)-(T − T_m_) function: (**a**) BT; (**b**) BST1; (**c**) BST2; (**d**) BST3; (**e**) BST4; and (**f**) BST4.5 ceramics.

For ferroelectrics with diffuse phase transition, a modified Curie–Weiss law has been proposed [40]:

(2) $$\varepsilon^{-1}-\varepsilon_{m}^{-1}={\left(T-T_{m}\right)}^{\gamma }\cdot C^{*-1}$$

where ε_m_ is the value of electric permittivity in maximum (at T_m_) and the exponent γ is a measure of the diffuseness of the phase transition and lies between 1 and 2 (γ = 1 for normal ferroelectrics, while γ = 2 for ideal relaxors). γ, obtained from the slope of the fitted straight line in Figure 7, increases with increases in ST content, suggesting that the material gradually shows relaxor-like behavior (Table 3), while C* is constant. Note that a linear fit is not satisfactory over some temperature ranges near T_m_, however, it is satisfactory over all temperature ranges after E-poling. In addition, γ decreases after E-poling. These observations suggest that prior electric field poling decreases the disorder introduced by the introduction of Sr.

**Figure 8:**
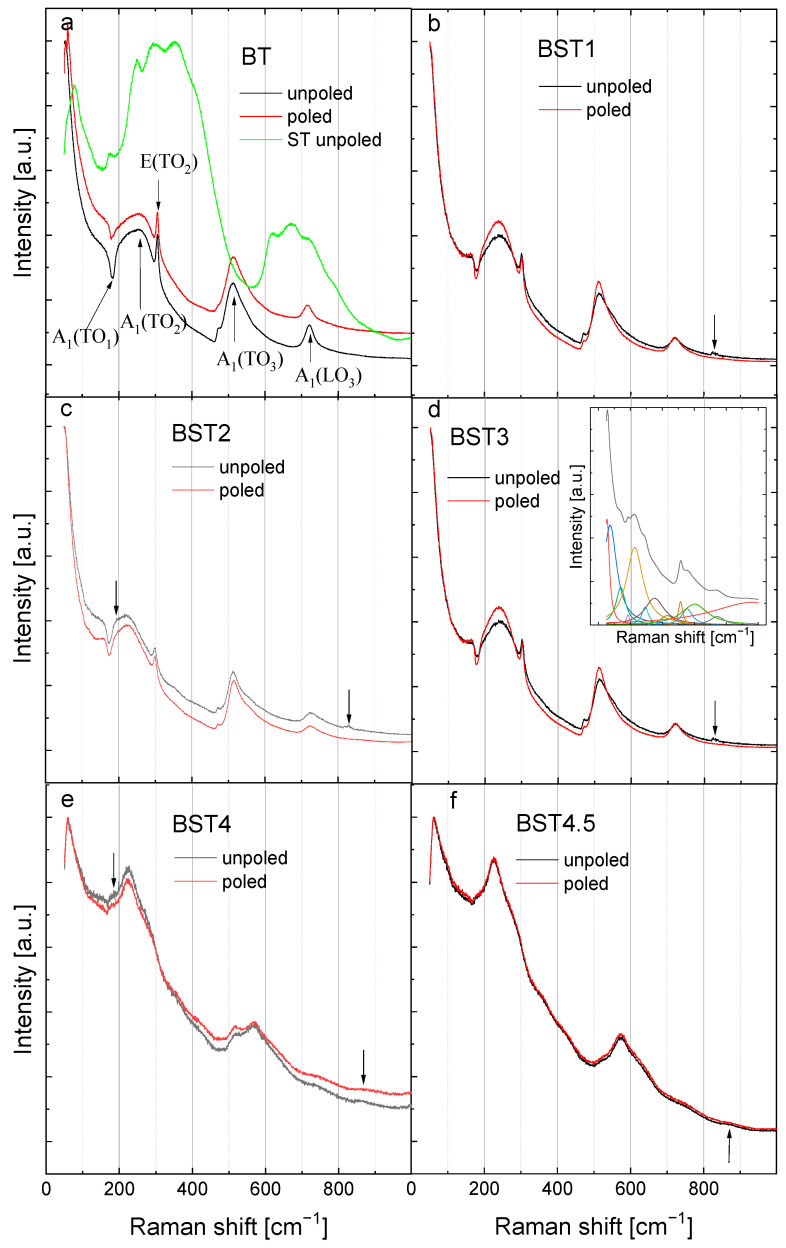
Room-temperature Raman spectra for: (**a**) BT; (**b**) BST1; (**c**) BST2; (**d**) BST3; (**e**) BST4; and (**f**) BST4.5 ceramics. The main bands for BT are indicated by arrows together with their assignment [43]. The inset shows, as an example, the spectral deconvolution of the room-temperature Raman spectrum of BST3.

Room-temperature Raman spectra of BT, ST and Ba_1−x_Sr_x_TiO_3_ are shown in Figure 8. In general, these spectra are assigned as belonging to the P4mm phase (except BST4 and BST4.5). The spectra can be de-convolved into fourteen peaks using Lorentzian functions (insert of Figure 8). The Raman spectrum of undoped BT shows a sharp peak centered around 55 cm^−1^ (A_1_(TO_1_)); a wide band centered around 270 cm^−1^ (A_1_(TO_2_)); a sharp peak at 308 cm^−1^ (A_1_ + E(TO + LO)); a broad peak at ~520 cm^−1^ (A_1_(TO_3_)); and a broad peak at ~720 cm^−1^ (A_1_(LO_3_)) in accordance with the results presented earlier [41,42]. In addition, a dip exists near 180 cm^−1^ (A_1_(TO_1_)). Both the dip near 180 cm^−1^ and a sharp peak at about 308 cm^−1^ are signatures of a ferroelectric state. Conversely, both a broad peak at about 270 cm^−1^ and a broad peak at about 720 cm^−1^ are characteristic of tetragonal symmetry. Pure ST displays two broad bands at about 300 and 650 cm^−1^, along with weak bands at about 156 and 185 cm^−1^. The bands can be assigned to second-order Raman scattering. Some changes in Raman spectra after ST introduction to BT are visible, although their features remain the same for x = 0.1–0.3. A few distinguished bands for Ba_1−x_Sr_x_TiO_3_ can be observed, the first one centered at 180 cm^−1^ (a dip indicating the ferroelectric phase), which is attributed to Ba–O vibrations, as in the mother BT phase. With the incorporation of Sr^2+^ ions, this mode seems to be diminished, with a corresponding change in the intensity.

The second wide band, centered around 270 cm^−1^, was attributed to the TiO_6_ octahedral vibration and to the existence of tetragonal symmetry in BT. This peak shifts toward lower wavenumbers, and their intensity decreases as the Sr^2+^ content increases, which can be attributed to the displacement of Ti and/or to the distortion of the oxygen octahedral (evolution of tetragonal symmetry). The sharp peak at about 308 cm^−1^ is broadened, and for x > 0.4, it nearly disappears. As this peak is commonly regarded as a sign of the long-range ferroelectric (tetragonal) ordering in pure BT, its disappearance indicates gradual loss of the ferroelectric state and a transition from tetragonal symmetry to cubic. The intensity of the band centered at about 520 cm^−1^ was observed to decrease. However, the intensity of the mode centered around 720 cm^−1^ (characteristic of the tetragonal phase) decreased gradually with increasing Sr^2+^ concentrations, which suggests a decrease in tetragonality in accordance with the X-ray diffraction results. Another important point to note is the extra small band at about 180 cm^−1^ (indicated by arrows for x = 0.2 and 0.4), which was previously observed for the BaTiO_3_-BiYbO_3_ system when the crystal symmetry changed from tetragonal to cubic [44]. Moreover, a new band appears (indicated by arrows) at about 830 cm^−1^ for x ≥ 0.4, the intensity of which seems to increase with higher dopant concentrations. This mode can also be associated with the average crystal symmetry changing from tetragonal to cubic. However, both modes are omitted in further descriptions due to their weak intensity and the poor reliability of their fitting results. Although symmetry is cubic for the compositions with x = 0.4 and 0.45, sharp peaks of the first-order Raman scattering exist in the background of the second-order Raman scattering. This seems to exclude the possibility of describing the symmetry by the Pm3m space group. Hence, the only possibility is that this high-temperature Raman activity originates from polar regions with a symmetry different than Pm3m. This is in accordance with the results of the measurements of the X-ray diffraction and ferroelectric properties (see below). It is reasonable to assume that the symmetries of the polar regions are the same as the ones which appear below the phase transition, i.e., P4mm. In general, the obtained Raman spectra of the BST ceramics are similar to those presented earlier for single-crystal BST [30]. If our prediction that the origin of the high-temperature Raman scattering is connected with the presence of polar regions is correct, it would indicate that these regions appear at higher temperatures than those in which departures from the Curie–Weiss law are observed.

**Figure 9:**
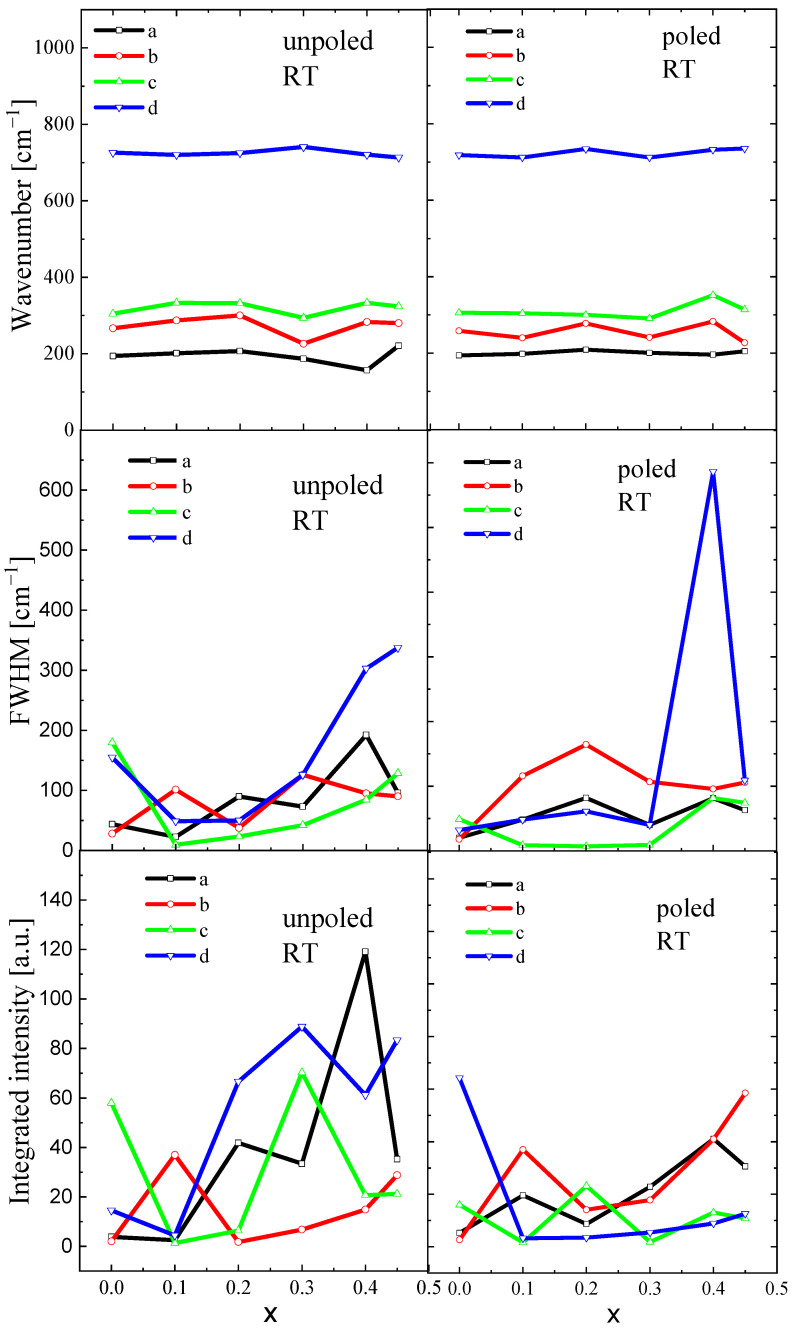
Compositional evolution of wavenumbers, FWHM and integrated intensity of BST ceramics. The a, b, c and d are mode names.

The spectra are similar for both unpoled and poled states, suggesting that E-poling does not significantly disturb the crystal structure. However, careful observation reveals some differences in the spectra. For undoped BT, E-poling leads to a decrease in the intensity of the bands and slightly shifts both, a dip at about 180 cm^−1^ and a band at about 720 cm^−1^, to higher wavenumbers, indicating an evolving tetragonal symmetry and/or ferroelectric state. However, the poled samples modified with Sr exhibit sharper, more symmetric and higher-intensity bands compared to the unpoled ones, suggesting that the degree of ordering becomes higher after E-poling. It is expected that the application of an electric field causes changes in ion distances and in the ion displacements, leading to some modification of the crystal structure. Changes in inter-ionic distances led to changes in force constants and, finally, to changes in vibrational mode frequencies. To further elucidate the local structural state of BST, the compositional dependence of mode wavenumbers, full widths at half maximum (FWHM) and integrated intensity-peak position are illustrated in Figure 9. In this investigation, statistical analysis methods were adopted to analyse the measurement errors of different parameters: peak positions, FWHM, and integrated intensity. The standard deviations were calculated as the square root of variance by determining each data point’s deviation relative to the mean. The analyzed Raman parameters were obtained with an accuracy of 2–6%.

Here, we focus on the selected modes, which are sensitive to the change in tetragonal-cubic symmetry, i.e., 180 (deep), 270, 308, and 720 cm^−1^ (see also Figure 8). As mentioned above, the first two modes are signatures of the ferroelectric phase, while the 308 and 720 cm ^−1^ are specific to tetragonal symmetry.

The wavenumbers of *a*, *b*, and *c* were shifted to a higher frequency up to x = 0.2 (optical phonons experience local hardening at x = 0.2); however, the wavenumber *d* was slightly shifted to a lower frequency up to x = 0.2, showing local softening. In contrast, damping (FWHM) of all modes slightly increased, showing some anomalies at x = 0.2. Integrated intensity of the modes changed irregularly without any clear tendency. These observations are a clear indication of Sr-induced phase transformation and seem to agree well with X-ray diffraction measurement data. It is probable that the observed characteristic anomaly of the wavenumber and FWHM at x = 0.2 can indicate the change in local symmetry from tetragonal P4mm to cubic Pm3m. Note that both crystal cell volume and tetragonality significantly evolved with increases in Sr content (see Table 2). E-poling leads to a levelling of the compositional dependence of wavenumbers, damping, and integrated intensity, retaining the tendencies characteristic of the unpoled state. In addition, the anomaly of the wavenumber, FWHM and integrated intensity at x = 0.2 is better visualized.

**Figure 10:**
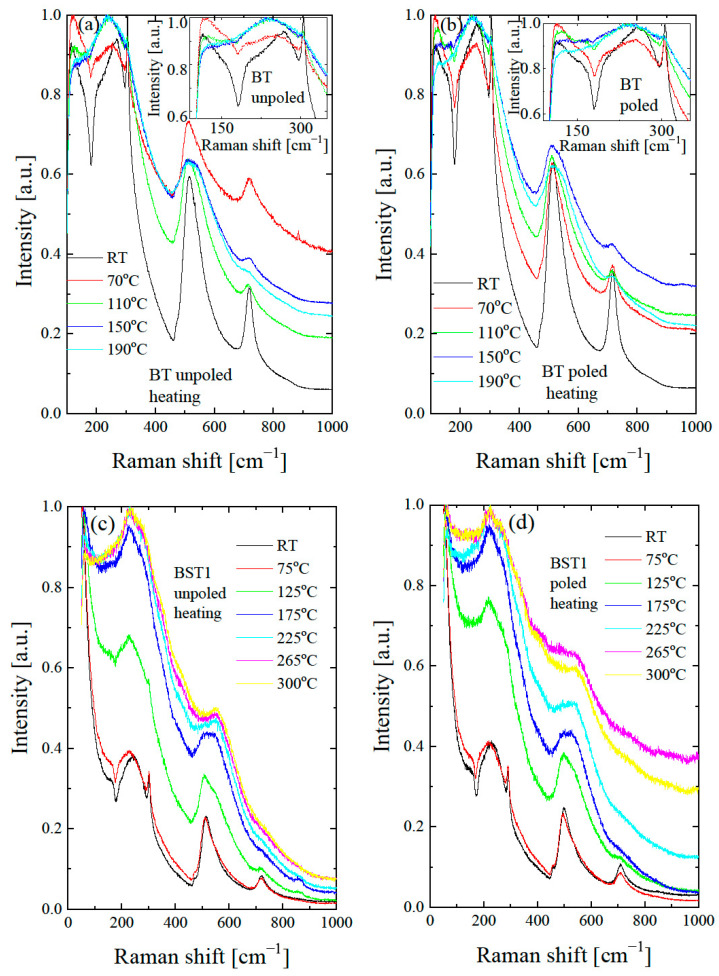

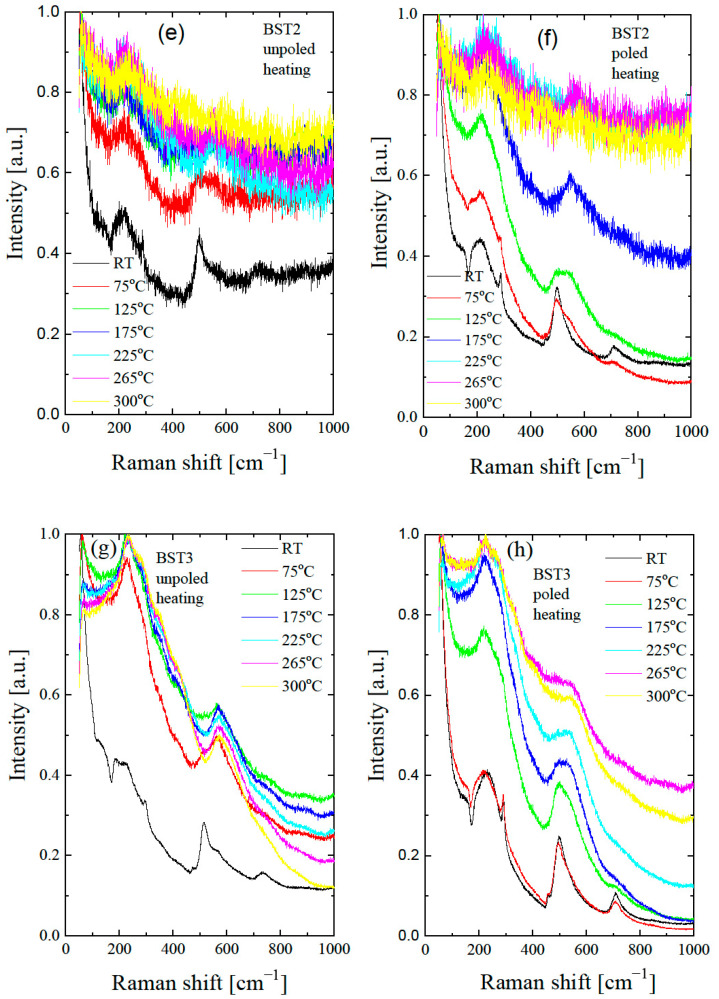

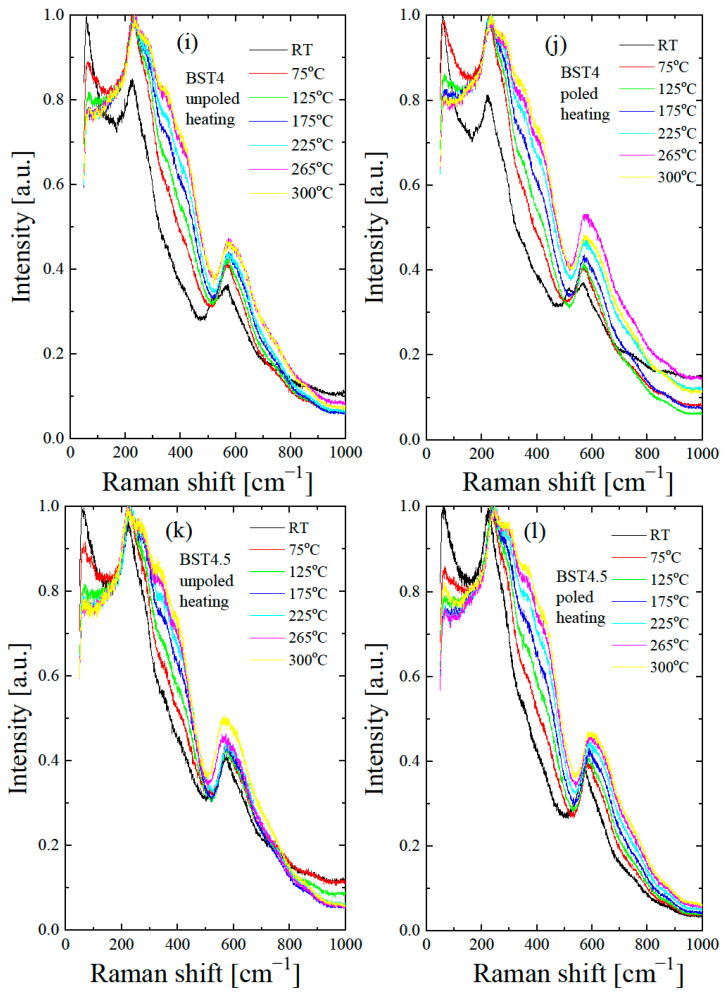
Thermal evolution of Raman spectra of BST for unpoled (**a**,**c**,**e**,**g**,**i**,**k**) and poled (**b**,**d**,**f**,**h**,**j**,**l**) states. Insets in Figure 10a,b show the expanded parts of these figures.

To investigate the thermal evolution of the local structure, temperature-dependent Raman spectra were collected for undoped BT and for Ba_1−x_Sr_x_TiO_3_ (Figure 10). It can be seen that the Raman modes for undoped BT become broader with increases in temperature, their intensity gradually decreases, and some modes disappear at the temperature of the tetragonal-cubic phase transition. The sharp peak at about 308 cm^−1^, the broad peak at about 720 cm^−1^ and the dip near 180 cm^−1^ gradually decreases and nearly disappears. In addition, the intensity of the 520 cm^−1^ band significantly decreases.

According to our X-ray diffraction and dielectric measurement results, the tetragonal to cubic transformation occurs at about 125 °C. However, two broad bands at 200–400 cm^−1^ and a weak, broad peak at 720 cm^−1^ persist even at 190 °C (see insert of Figure 10a), which may occur as a result of the polar nanoregions existing in the cubic matrix, in accordance with the XRD results (see also below). The split in this band and the existence of a 720 cm^−1^ peak are usually found in tetragonal perovskite materials [45]. The splits in the 200–400 cm^−1^ band, and the 720 cm^−1^ feature are more distinguished and exist over a higher temperature range after the action electric field in comparison with the unpoled state (see insert in Figure 10b). The temperature-dependent Raman spectra of Ba_1−x_Sr_x_TiO_3_ for x = 0.1–0.3 show similar changes with increases in temperature, as in the case of undoped BT. The bands at 305, 520, 720 and the dip at ~180 cm^−1^ gradually lose intensity with increases in temperature. According to our X-ray diffraction and dielectric measurement results, the tetragonal to cubic transformation occurs at about 130, 110, 80, 35, 20 and 15 °C for BT, BST1, BST2, BST3, BST4 and BST4.5, respectively (Table 1). However, the band at about 720 cm^−1^ persists even at 300 °C (though as a very broad one) for all BST samples. Additionally, the splits of the band 200–400 cm^−1^ could be distinguished at temperatures far above 200 °C for all BST samples, which hints the local tetragonal symmetry in this temperature range. The main effect of E-poling is a shift in the previously mentioned changes in the ~180, ~200–400, ~308 and ~720 cm^−1^ bands to higher temperatures, which indicates that the electric field supported a tetragonal (ferroelectric) phase. BST4 and BST4.5 show the small temperature evolution of the Raman spectra in both unpoled and poled states. Note that, according to the X-ray diffraction results, both samples have cubic symmetry over the investigated temperature range. The existence of two very broad peaks at 200–400 cm^−1^ and 500–650 cm^−1^, which are evidence of the cubic phase of both samples, confirms these results. Despite this, both samples show first-order Raman spectra, which implies that this phase does not have perfect cubic symmetry (some content of the tetragonal phase in the cubic matrix exists), in accordance with the XRD results.

**Figure 11:**
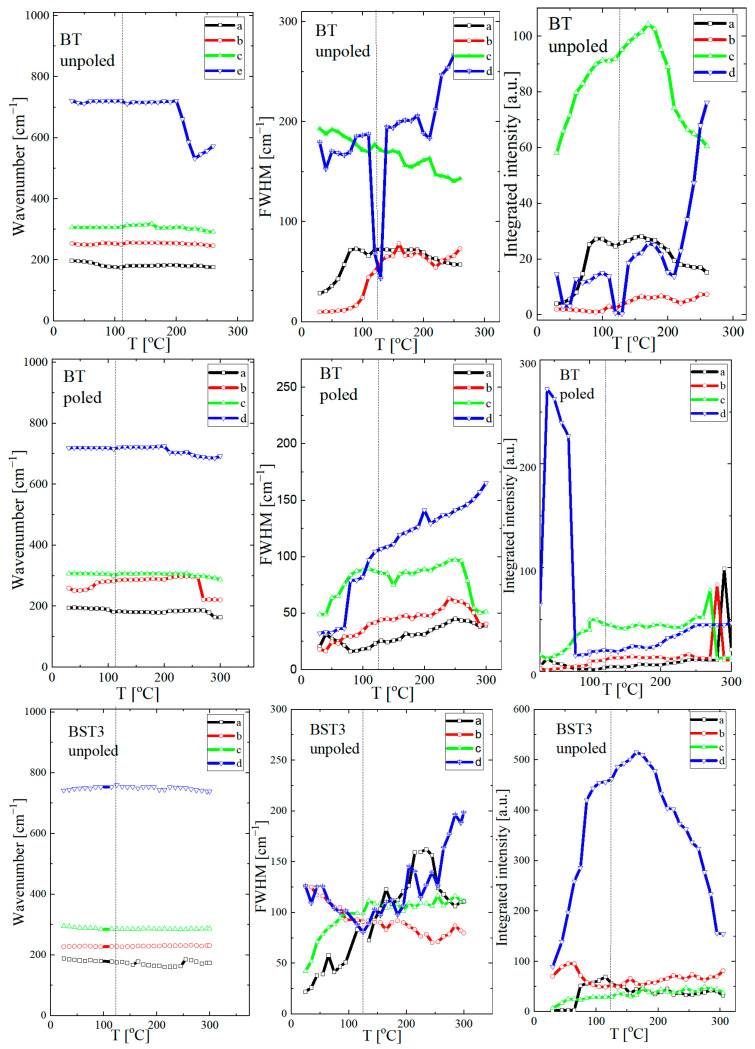

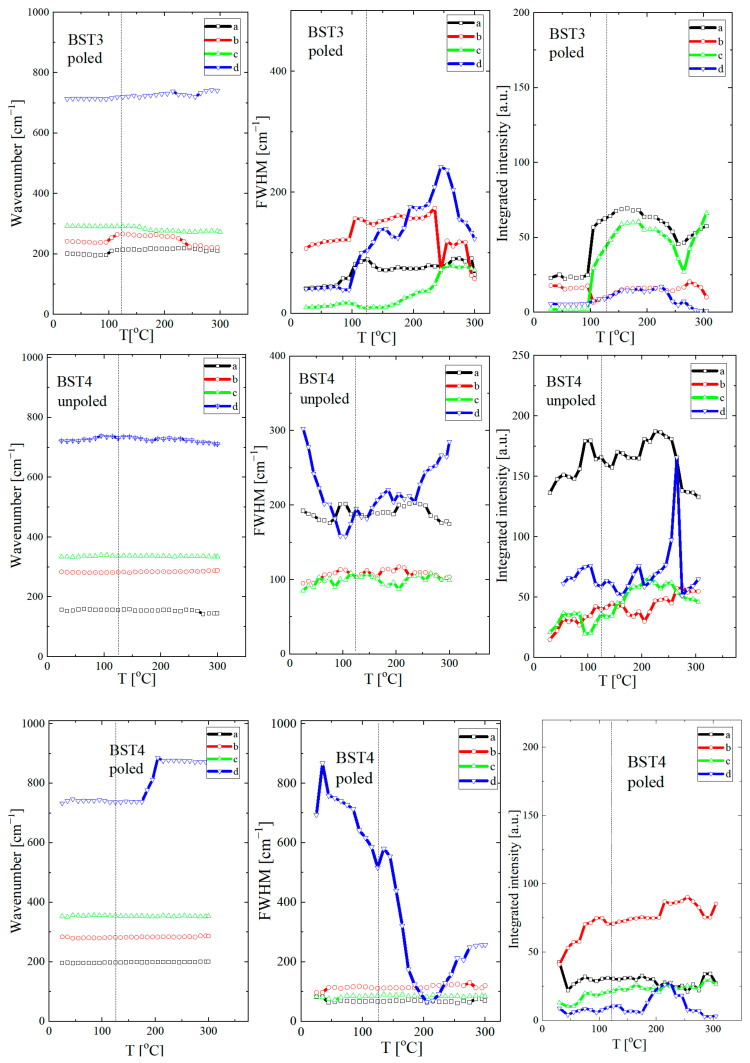
Temperature evolution of wavenumbers, FWHM, and integrated intensity of undoped BT and BST3 and BST4 ceramics. On each figure, the points are fitting parameters after deconvolution (see inset in Figure 8d).

For a closer inspection, the temperature variations of the peak position, line width, and intensity of the modes for compositions BT, BST3 and BST4 (BST1, BST2 and BST4.5, see Supplementary Materials Figure S2) are presented in Figure 11 (the same symbols are used for particular modes, as for Figure 9). The tetragonal-cubic transition temperature in undoped BT is also marked. It is expected that discontinuous changes in the Raman peaks can reveal signs of local transition. As can be seen, the modes alter non-monotonically, with a sudden change around the tetragonal-cubic phase transition. This includes a significant increase in the intensity of the peaks and an abrupt jump in their location and width; this can be related to the different ionic arrangements associated with the change in crystal symmetry. The lines show a similar evolution in the temperature dependence of integrated intensity, namely, the intensities achieve the maxima or minima near the phase transition temperature. These features can be related to changes in the crystal structure and domain structure, which influence the optical properties. In general, the line widths (FWHM) show an almost classical temperature evolution, i.e., they increase with increases in temperature and show an anomaly near the phase transition temperature. The temperature evolution of the line width is attributed to anharmonic effects [46,47]. In the case of BST, the temperature evolution of this parameter is mainly determined by chemical disorder in the A-site and static and/or dynamic local inhomogeneities related to the off-centred ion displacements correlated on different scales. The changes in the Raman line parameters are more visible for poled samples, which suggests a transition from the electric-field-induced ferroelectric state previously destroyed by the introduction of ST.

According to Petzelt et al. [48], the Raman strength (integrated intensity) of polar modes is proportional to the average polarisation or total volume of the polar regions. Generally, the integrated intensity of Raman modes slightly increases with increases in temperature, as can be seen from Figure 11. This evolution of the integrated intensity of Raman modes is correlated with the temperature of the tetragonal-cubic phase transition of the samples, c. 125 °C for undoped BT. This parameter decreases when the cubic phase dominates in the higher temperature region (>200 °C). Similar features are observed for BST1, BST2 and BST3 samples. However, the evolution of this parameter is observed in the low-temperature range due to a decrease in the temperature of the tetragonal-cubic phase transition linked to increasing Sr content. For the BST4 and BST4.5 samples, irregular changes in this parameter are observed. According to Table 1, we expected the phase transition to be below room temperature for both samples. The irregular changes in integrated intensity can be explained by the appearance of polar regions, which decrease gradually with increases in temperature owing to the decrease in the correlation length in ion displacement, and the change from a stable to an unstable state.

The FWHM of the Raman peaks are used as indicators of crystal quality [49]. The FWHM of the majority of the modes slightly increased or stabilised, which suggests that the samples become more symmetric at local scales with increases in temperature.

The Raman scattering confirms the fact that the crystal structure of BST, as determined from the XRD studies, is only an average.

Although it is difficult to precisely interpret the obtained Raman scattering data, changes on a local scale are clearly visible, as well as their influence on macroscopic properties. Due to this, only an approximate estimation of the tetragonal to cubic phase transition is possible from the Raman scattering data. This estimation was made based mainly on the clear anomaly of the temperature dependence of the wavenumber, line width, and integrated intensity. The estimation gives the following temperatures for the tetragonal-cubic phase transition: ~120, ~95, ~65 and ~30 °C for x = 0, 0.1, 0.2 and 0.3, respectively (Table 1).

**Figure 12:**
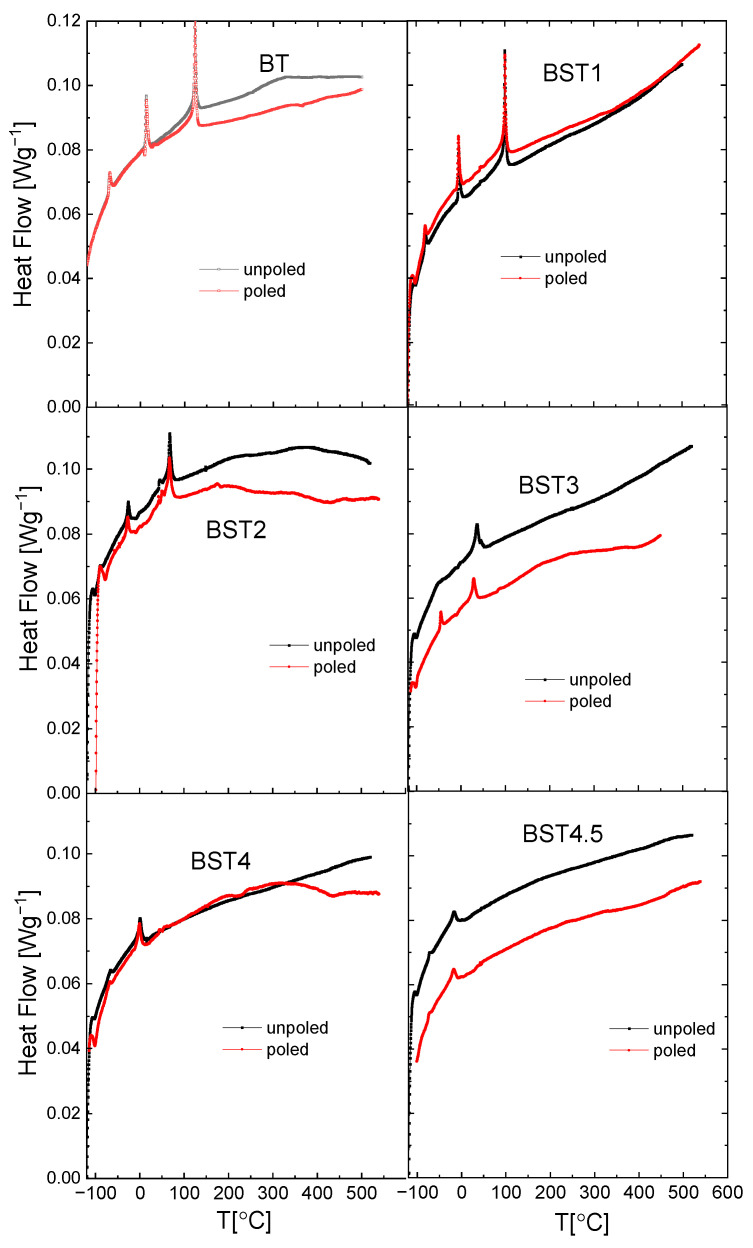
Temperature dependences of heat flow recorded during heating and cooling processes for BT, BST1, BST2, BST3, BST4, and BST4.5 ceramics.

Figure 12 presents the results of DSC (differential scanning calorimetry) analysis of the Ba_1−x_Sr_x_TiO_3_ ceramics. We can see that the peaks related to three phase transitions in undoped BT become smaller (decreased area under the peaks) and shift to a lower temperature with a rise in ST content. As those areas represent a free-energy difference between the two phases, this indicates that the addition of ST decreases the stability of both phases. In addition, these peaks become broader (the temperature width of phase transitions estimated from the onsets and ends of DSC peaks increases). Particularly, the peaks related to the rhombohedral-orthorhombic phase transitions for x = 0.4 and 0.45 are very broad. The temperatures of the DSC and electric permittivity anomalies coincided well, as is shown in Figure 4 and Figure 12, respectively (see also Table 1). In general, the temperatures of DSC anomalies shift towards higher temperatures after E-poling. In addition, the DSC peaks are sharper for poled samples in comparison to unpoled ones. These suggest that the electric field seems to reconstruct the structural disorder caused by the introduction of Sr to BT.

**Figure 13:**
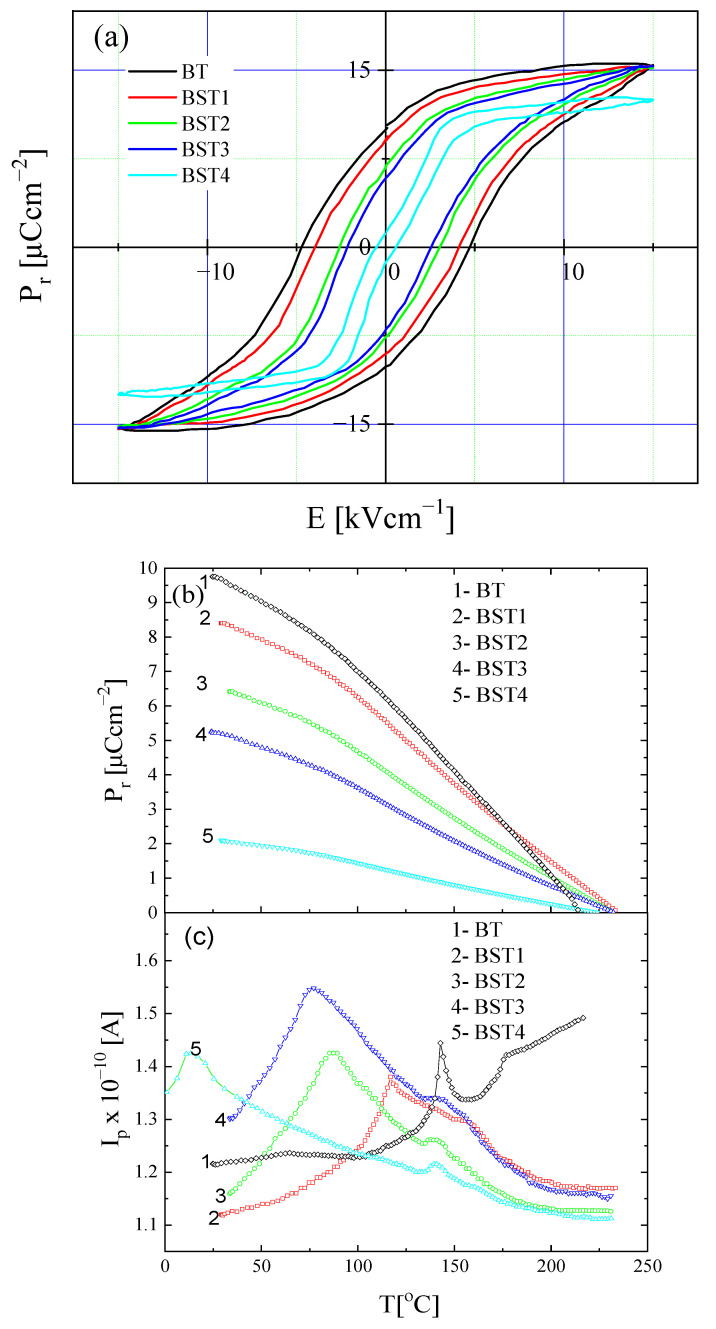
Hysteresis loops of BT, BST1, BST2, BST3, BST4 ceramics at room temperature (**a**); temperature evolution of remnant polarization P_r_ (**b**); and pyroelectric current (**c**) of BT, BST1, BST2, BST3, BST4 ceramics.

To investigate the effect of the ST addition on the ferroelectric properties of (1-x)BT-xST system, hysteresis loops and pyroelectric measurements were performed (Figure 13). For x = 0, the loops displayed saturated ferroelectric behavior with P_r_ = 10 μCcm^−2^ and E_c_ = 5 kVcm^−1^ (Figure 13a). A small amount of ST (x = 0.1) leads to a small decrease in P_r_ to 8.6 μCcm^−2^ and E_c_ to 4.1 kVcm^−1^. P_r_ decreases to 6.5 μCcm^−2^ and E_c_ decreases to 2.4 kVcm^−1^ for x = 0.3. With further increases in ST content, both P_r_ and E_c_ decrease. Thus, P_r_ and E_c_ were depressed with increases in ST, leading to slim loops, which is characteristic of a relaxor-like state. As can be seen from Figure 13b, P_r_ obtained from pyroelectric measurements decreases with increases in ST content, in accordance with the hysteresis loops measurement. The remnant polarization is retained above the Curie temperature and an additional small anomaly exists in the pyroelectric current i(T) curve above the Curie temperature (Figure 13c), indicating the existence of residual domains/polarization in this temperature range, even for undoped BT, in accordance with the present X-ray diffraction and Raman results. As mentioned, the existence of stable polar nanoregions in the cubic phase of even undoped BT was suggested by both the theoretical and experimental results [12,13,14,15,16]. Slim loops and remnant polarization exist even for x = 0.4. This is due to the coexistence of cubic and tetragonal phases near room temperature, which favors the inducing of a ferroelectric-like state by the electric field used in the experiments.

**Figure 14:**
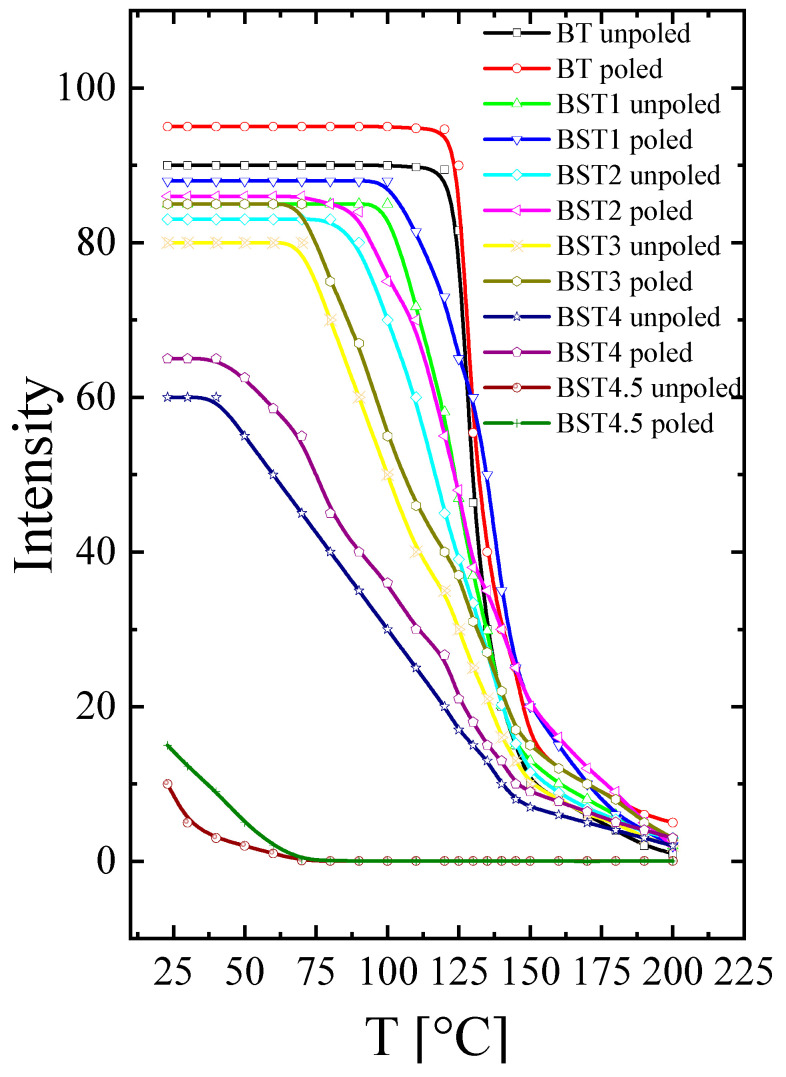
Temperature evolution of SHG intensity of unpoled and poled BT, BST1, BST2, BST3, BST4, BST4.5 ceramics.

The temperature evolution of SHG intensity for the unpoled and poled samples is presented in Figure 14. As can be seen, the SHG(T) curve shows high intensity for undoped BT, and this intensity decreases with increases in Sr content. A weak SHG intensity still exists over a temperature range far above the tetragonal-cubic phase transition, even for undoped BT, which suggests that the cubic phase is non-centrosymmetric in this temperature range. This is in accordance with earlier results [12,13,14,15,16]. A similar effect is observed for the BT–ST system. The BST4.5 ceramic has a weak, nonzero value of SHG intensity over a wide temperature range, which implies that the cubic phase has some disorder (i.e., is not purely centrosymmetric for this material). The non-centrosymmetry detected within the cubic phase of the investigated system may be attributed to polar micro- and nano-regions dispersed in a centrosymmetric matrix, which is consistent with the results of the X-ray diffraction, Raman, and ferroelectric behaviour measurements. E-poling leads to a slight increase in SHG intensity for undoped BT and a higher increase for BT–ST. This is because the ferroelectric order is strong for undoped BT and weaker for the BT–ST system, and the electric field enhances the ferroelectric order destroyed by Sr-doping BT. Additionally, E-poling expanded the low-temperature temperature-independent SHG intensity range to higher temperatures, implying that the long-range ferroelectric phase is sustained by the electric field. The temperature of the tetragonal-cubic phase transition of BST was estimated as the temperature at which the SHG signal starts to decrease. This estimation gives the following phase transition temperatures: ~125, ~100, ~75 and ~35, for x = 0, 0.1, 0.2 and 0.3, respectively.

**Figure 15:**
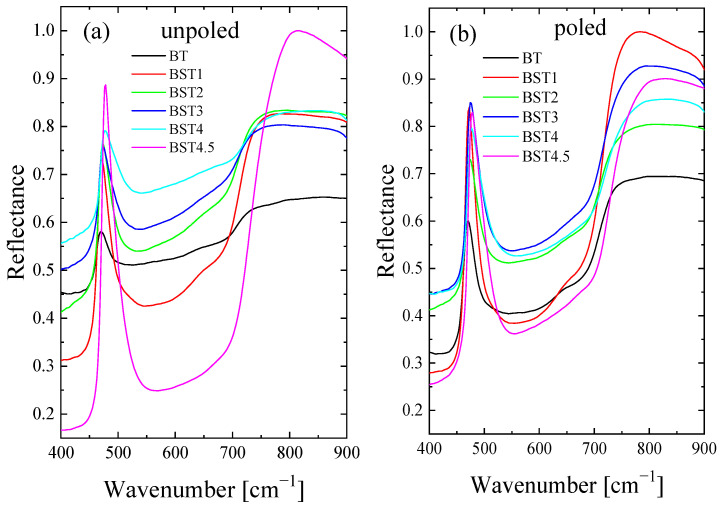
Infrared reflectivity spectra of: (**a**) unpoled; and (**b**) poled BT, BST1, BST2, BST3, BST4, BST4.5 ceramics.

The room-temperature FTIR spectra of the Ba_1−x_Sr_x_TiO_3_ ceramics in the wavelength interval 170–900 cm^−1^ are shown in Figure 15. The three reflection bands at 150–400, 400–600 and 600–800 cm^−1^ are visible. The lowest bands characteristic of undoped BT lie below our experimental conditions. A visual examination reveals some evolution of the spectra and a slight shift towards higher wavenumbers with increases in Sr content, although their features remain the same. This shift can be explained mainly by the smaller ionic radius of strontium relative to the barium occupying the A-site, resulting in decreased unit cell volume. Peaks corresponding to undoped BT become less prominent with the increase in ST content. A significant change is experienced by the modes grouped in the vicinity of 100–200 cm^−1^ associated with the A-site ion-TiO_6_ vibrations. The 400–600 cm^−1^ band is assigned to Ti-O vibrations. The 600–800 cm^−1^ band is related to the stretching vibration of the TiO_6_ octahedra. The decrease in unit cell volume enhances interactions between both A-site ions and TiO_6_ octahedra, and between Ti and O ions, which leads to shifts in the corresponding bands in the FTIR spectrum to a higher frequency. The mode at about 320 cm^−1^ becomes gradually broader and disappears for x ≥ 0.4, and is related to the evolution of, and finally, to the disappearance of, tetragonal symmetry. In general, poled samples exhibit sharper and more symmetric bands than unpoled ones, which again indicates that the electric field increases the structural order previously destroyed by Sr-doping. Some redistribution of the intensity of the bands is also visible. In addition, E-poling leads to a shift in the bands toward a higher wavenumber (5–8 cm^−1^). These differences can be helpful for accurate studies of poling and depoling processes for these materials (in other words, the differences can be used to evaluate the efficiency of the poling and/or depoling process [50]).

**Figure 16:**
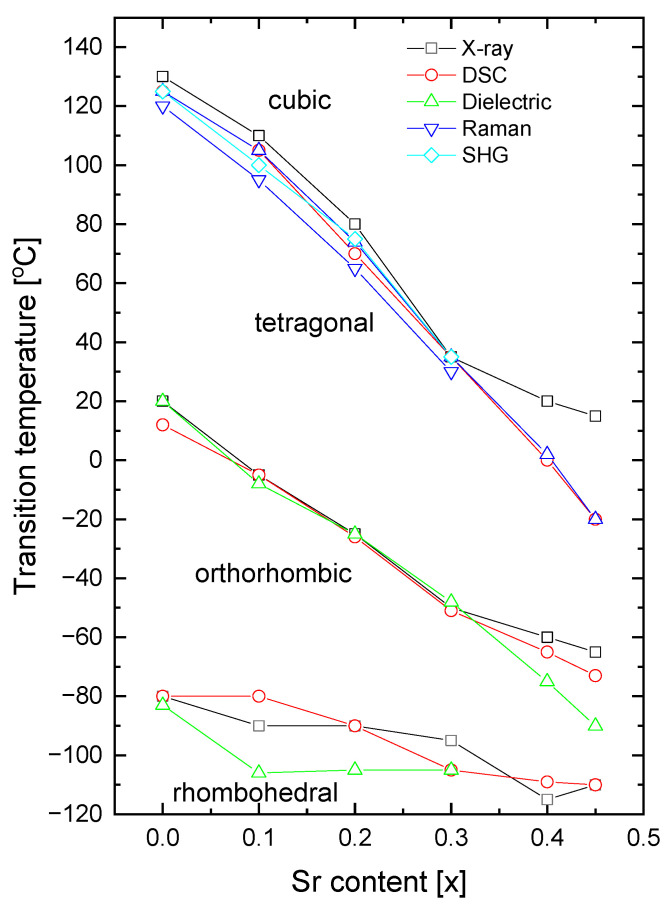
Sr-composition dependence of transition temperatures for rhombohedral-orthorhombic (T_R-O_); orthorhombic-tetragonal (T_O-T_); and tetragonal-cubic (T_T-C_) of BT, BST1, BST2, BST3, BST4, BST4.5 ceramics.

The transition temperatures obtained from our measurements are plotted against the Sr content in Figure 16. Excellent agreement between the transition temperatures, obtained using XRD diffraction, dielectric, and DSC measurements, is clearly evident. An important point to note is the lower temperature of the tetragonal to cubic phase transition, as obtained from Raman scattering, in comparison with those obtained by studying the X-ray diffraction, DSC, and dielectric measurements. This difference can be partially caused by stress effects. As shown in previous papers [51,52], the phonon frequencies can shift remarkably due to the existence of stresses in the material. In fact, the ionic radius of Sr (r_Sr2+_ = 1.44 Å with coordinator number XII) is smaller than that of Ba (r_Ba2+_ = 1.61 Å with coordinator number XII) [53] and compressive stress is expected. This compressive stress can shift the temperature of the tetragonal-cubic phase transformation towards lower temperatures [51,52]. As the shift is not significant, this suggests rather uniform incorporation of Sr ions into BT without the introduction of distinct mechanical stress. As can be seen from the figure, an almost linear decrease in the Curie point with an increase in Sr content was observed (see also Table 1), in accordance with earlier results [54,55].

**Figure 17:**
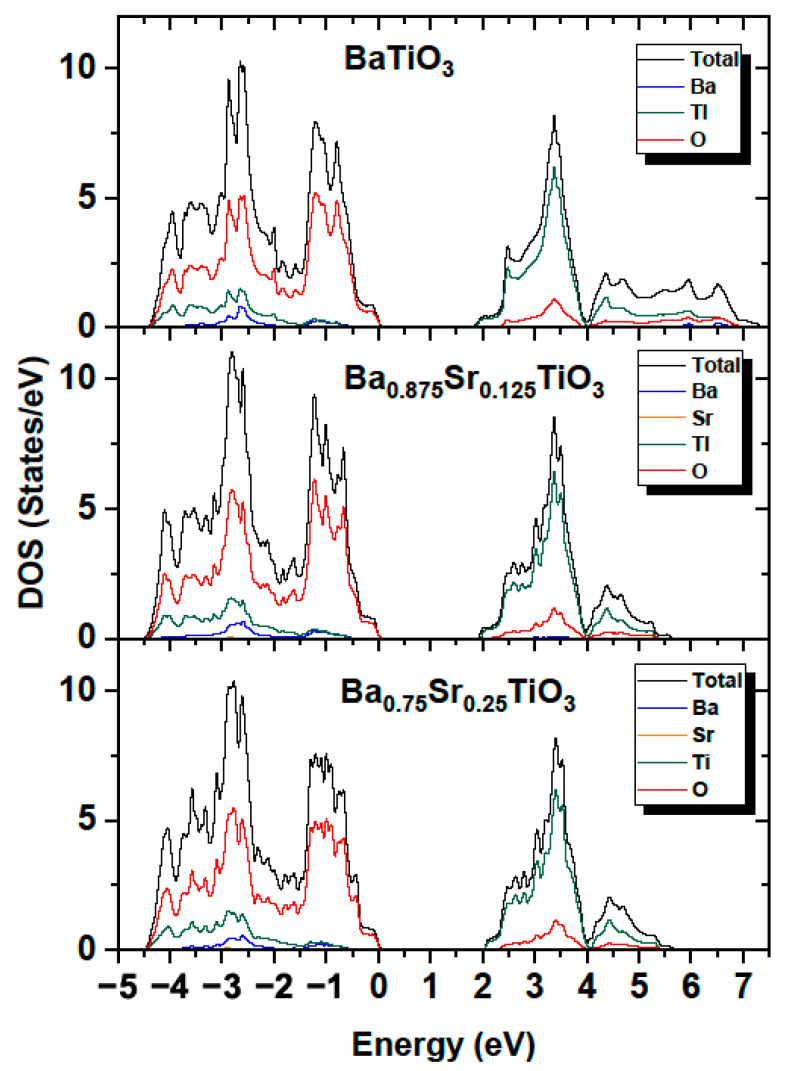
Total and partial density of states in BaTiO_3_; Ba_0.875_Sr_0.125_TiO_3_; and Ba_0.75_Sr_0.25_TiO_3_.

The partial (PDOS) and total (TDOS) densities of the states of Ba_1−x_Sr_x_TiO_3_ systems were computed for concentrations x = 0, 0.125 and 0.25. Figure 17 shows the calculated total and partial densities of electronic states for BaTiO_3_, Ba_0_._875_Sr_0.125_TiO_3_, and Ba_0.75_Sr_0.25_TiO_3_. The conduction band (CB) in the range of 1.93–5.7 and 2.04–5.7 eV for compounds containing strontium consisted mainly of Ti (3d) orbitals and to a lesser extent of O (2p) states. The conduction band in the case of BT is wider and is in the range of 1.77–6.8 eV. For comparison, in article [56] this range started at 2.7 eV. The dominant contribution at the top of the conduction band originated from Ti (3d) electrons. The highest hybridization in the CB was observed in the middle region of the band formed by Ti (3d) and O (2p) states. The valence band (VB) is wider than the conduction band (CB) for Ba_0.875_Sr_0.125_TiO_3_ and Ba_0.75_Sr_0.25_TiO_3_, and was calculated to be 4.5 eV. The valence band consisted mostly of O (2p) states. Hybridization also occurred in the lower energy section of the VB and originated from the O (2p), Ti (3d) and Ba (4p) orbitals. The calculations show that in the Sr-doped compound, the valence band grew wider (Figure 17).

**Figure 18:**
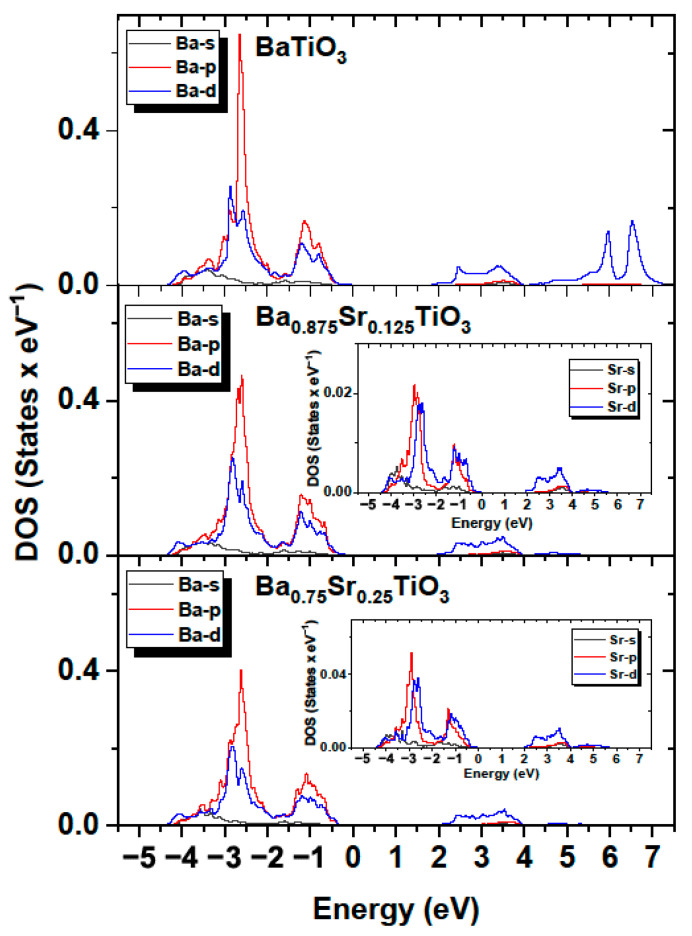
Partial density of states in BaTiO_3_; Ba_0.875_Sr_0.125_TiO_3_; and Ba_0.75_Sr_0.25_TiO_3_ for Ba and Sr (inset) atoms.

The influence of the components forming the VB and CB bands was similar to that observed for BT. As in the case of BT, the contribution from Ba orbitals was relatively small. Moreover, the contributions from the valence states of Ba to the occupied bands were small, since the barium atoms present in the crystal occurred in the Ba^+^ ionic state. The contribution from Sr orbitals was negligible. The results shown in Figure 18 confirm that the valence band was formed mainly by oxygen atoms, while the conduction band was determined by the titanium atoms for both Ba_0.875_Sr_0.125_TiO_3_, Ba_0.75_Sr_0.25_TiO_3_ and BaTiO_3_. The calculated bandgaps were 1.77, 1.94 and 2.05 eV for BaTiO_3_, Ba_0.875_Sr_0.125_TiO_3_, Ba_0.75_Sr_0.25_TiO_3_, respectively. An increase in the bandgap may lead to an escalation in the insulation capabilities, giving rise to a high breakdown strength.

Figure 18 shows the calculated partial densities of electronic states for BaTiO_3_, Ba_0.875_Sr_0.125_TiO_3_ and Ba_0.75_Sr_0.25_TiO_3_ for barium and strontium atoms. It can be observed that both the conduction and valence band were determined by the p and d orbitals, while the contribution from s orbitals was relatively small. It can also be observed that, with increasing strontium doping, the bandgap increases (Figure 17). The upward shift in the lower conduction band towards higher energies can be explained as a result of the interaction of the lower of the conduction band with the Ti (3d) orbitals mixed with co-doping of the Sr (4p/d) states. It was determined that the bandgap for Ba_0.875_Sr_0.125_TiO_3_ and Ba_0.75_Sr_0.25_TiO_3_ is larger than that for BaTiO_3_ by 0.17 and 0.28 eV, respectively. The increase in the energy gap is linked by the fact that pure SrTiO_3_ has a d^0^ configuration, which makes it an insulator due to its band structure. Both barium and strontium have filled s subshells. This result can be explained by the fact that the experimental energy gap in SrTiO_3_ is 3.25–3.75 eV [57], which is greater than in the case of BaTiO_3_. For BaTiO_3_, this value is consistent with the experimental value of 3.2 eV [58]. It can therefore be concluded that doping BT with Sr atoms leads to an increase in bandgap. It should be noted that the calculations performed so far with various ab initio codes and methods indicate that the optical gap ranges from 2.31 to 3.96 eV [59] for SrTiO_3_.

**Figure 19:**
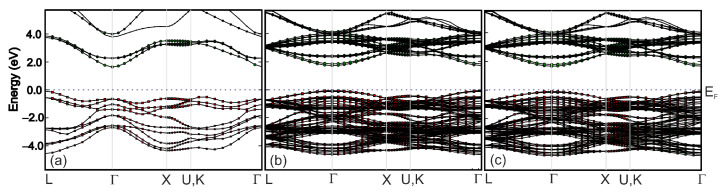
Computed (GGA) energy band structure of (**a**) BaTiO_3_, (**b**) Ba_0.875_Sr_0.125_TiO_3_ and (**c**) Ba_0.75_Sr_0.25_TiO_3_ along the high-symmetry directions in the Brillouin zone.

The calculated band structures of BaTiO_3_, Ba_0.875_Sr_0.125_TiO_3_, Ba_0.75_Sr_0.25_TiO_3_ compounds are compared in Figure 19. The band structures profiles of Ba_1−x_Sr_x_TiO_3_ are similar for various Sr concentrations x = 0.125 and 0.25. The difference in bandgap values between the valence and conductivity bands was due to differences in the composition of the cation atoms and the crystal structure of these three systems, which was a consequence of the substitution of barium ions with strontium ions. In addition, as can be seen from Table 4, the tetragonality (c/a) and volume of the unit cell (V) decreases with increasing amounts of Sr. The dependence changes in c/a and V are linear. A similar relationship was observed in our experimental data for lattice volume and tetragonal distortion when increasing the amount of ST (see also Table 2). The observed differences between the calculated and experimental values can be mainly interpreted as the result of internal stresses in the ceramics.

There are at least two possible reasons why the ferroelectric properties of BT weaken as a result of partial substitution of the A-site Ba^2+^ ions by Sr^2+^. The first one is an increase in the degree of disorder, which leads to a disturbance in the long-range ferroelectric state. As mentioned, the Sr^2+^ ion has a smaller ionic radius (1.44 Å) than Ba^2+^ (1.61 Å), which gives rise to compressive stress and, as a consequence, to a decrease in unit cell volume and a decrease in tetragonal distortion (see Table 1). Local elastic fields are thus expected. The decrease in polarization with the rise of ST (Figure 13) may be related to a decrease in tetragonal distortion (see Table 1). In addition, some local chemical disordering, characteristic of solid solutions, could also occur upon substitution, creating chemically ordered regions which can have polar properties (polar nanoregions) responsible for the increased frequency dispersion and the appearance of relaxor-like behavior. The second reason is a change in grain morphology upon substitution. The substitution of Sr^2+^ to BT leads to non-uniform grain sizes, which generate more internal stress, making movements in the domain walls difficult. This could lead to a decrease in polarization.

On the other hand, the incorporation of Sr^2+^ in Ba^2+^ can induce a stronger covalence due to the higher electronegativity of the former ion compared to the latter one (0.95 vs. 0.89). Thus, the nature of the Sr–O bond seems to be partially covalent, whereas the Ba–O bond is purely ionic. This can lead to a gain in ferroelectric behavior. As this behavior weakens, we can conclude that differences in the ionic radii of Sr^2+^ and Ba^2+^, chemical disordering, and changes in the grain morphology, are predominant. Thus, the present study shows that the properties can be tuned by both the incorporation of foreign ions and by electric field action.

## 4. Conclusions

Microstructure/structure, Raman scattering, infrared spectroscopy, dielectric, second harmonic generation (SHG) and ferroelectric studies of SrTiO_3_ (ST)-doped BaTiO_3_ (BT) ceramics in unpoled and poled states were performed. The X-ray diffraction and DSC data established that the tetragonal to cubic transition temperature decreases with increases in Sr content. The Sr-induced structural transformations were further confirmed by the Raman scattering results. Excellent agreement was revealed between the transition transformation temperatures obtained by X-ray diffraction, dielectric, and DSC measurement studies. The analysis of pyroelectric and hysteresis loops showed that the ferroelectric properties were depressed upon doping. The dielectric behavior evolves with increasing ST content from classical ferroelectric to relaxor-like, and this transformation is accompanied by a shift in the permittivity maxima towards lower temperatures. The polar state was disrupted into a disordered, almost relaxor-like state by ST-doping of BT. It is proposed that these effects are due to ionic size differences and an increase in the covalent character of the A–O bond, and that the former reason is dominant. SHG generation results indicate the presence of polar micro/nano-regions above the tetragonal-cubic phase transition. It was shown that electric field poling seems to reconstruct ordering disturbed by the introduction of ST into BT. Ab initio calculations showed that doping BaTiO_3_ with Sr atoms leads to an increase in the bandgap. Doping BT with strontium atoms leads to an upward shift in the lower conduction band towards higher energies.

## Figures and Tables

**Table 1:** Phase transition temperatures (rhombohedral-orthorhombic (T_R-O_), orthorhombic–tetragonal (T_O-T_), and tetragonal-cubic (T_T-C_) in °C) of Ba_1−x_ Sr_x_TiO_3_ ceramics through X-ray, DSC, dielectric, Raman, and SHG measurements. The accuracy of the measurements depends on the resolution of a measuring instrument.

	x = 0			x = 0.1			x = 0.2			x = 0.3			x = 0.4			x = 0.45		
	T_R-O_	T_O-T_	T_T-C_	T_R-O_	T_O-T_	T_T-C_	T_R-O_	T_O-T_	T_T-C_	T_R-O_	T_O-T_	T_T-C_	T_R-O_	T_O-T_	T_T-C_	T_R-O_	T_O-T_	T_T-C_
X-ray	−80	20	130	−90	−5	110	−90	−25	80	−95	−50	35	−115	−60	20	−110	−65	15
DSC	−80	12	125	−80	−5	105	−90	−26	70	−105	−51	35	−109	−65	0	−110	−73	−20
Dielectric	−83	20	125	−106	−8	105	−105	−25	74	−105	−48	35	-	−75	2	-	−90	−20
Raman	-	-	120	-	-	95	-	-	65	-	-	30	-	-	-	-	-	-
SHG	-	-	125	-	-	100	-	-	75	-	-	35	-	-	-	-	-	-

**Table 2:** XRD results of Ba_1−x_Sr_x_TiO_3_ ceramics, V is the unit cell volume, c/a is the tetragonal distortion.

Sample	Phase Composition	V (Å^3^)	c/a
BaTiO_3_	P4mm	64.43 (2)	1.0060 (1)
Ba_0.9_Sr_0.1_TiO_3_	P4mm	63.84 (1)	1.0035 (2)
Ba_0.8_Sr_0.2_TiO_3_	P4mm	63.24 (3)	1.0030 (2)
Ba_0.7_Sr_0.3_TiO_3_	P4mm	62.65 (1)	1.0024 (1)
Ba_0.6_Sr_0.4_TiO_3_	Pm3m	62.07 (2)	-
Ba_0.55_Sr_0.45_TiO_3_	Pm3m	61.66 (1)	-

**Table 3:** Exponent γ of the investigated samples.

Samples	γ	
	Unpoled	Poled
BT	1.091 (1)	1.052 (1)
BST1	1.112 (1)	1.071 (1)
BST2	1.111 (2)	1.101 (1)
BST3	1.122 (1)	1.092 (1)
BST4	1.183 (3)	1.133 (2)
BST4.5	1.272 (2)	1.241 (2)

**Table 4:** Ab initio simulation of Ba_1−x_Sr_x_TiO_3_: a, b—cell parameters, V—volume of unit cell and ratio c/a compared to experimental data.

Compound	a [Å]	c [Å]	c/a	Expr. c/a	V [Å^3^]	Expr. V [Å^3^]
BT	4.000955	4.220442	1.05486	1.0060	67.55932	64.43
Ba_0.875_Sr_0.125_TiO_3_	3.991119	4.193231	1.05064	1.0035 *	66.79411	63.84 *
Ba_0.75_Sr_0.25_TiO_3_	3.982019	4.168434	1.04681	1.0030 **	66.09668	63.24 **

* Ba_0.9_Sr_0.1_TiO_3_; ** Ba_0.8_Sr_0.2_TiO_3_.

## Data Availability

Data is contained within the article or supplementary materials. The data presented in this study are available on request from the corresponding author.

## Supplementary Materials

The following supporting information can be downloaded at: https://www.mdpi.com/article/10.3390/ma16186316/s1, Supplementary Figure S1: the average grain size as a function of Sr content. Figure S2: temperature evolution of wavenumbers, FWHM and integrated intensity of BST1, BST2, and BST4.5 ceramics. In each figure, the points are fitting parameters after deconvolution.
