# Supplementary material for: Temperature and E-Poling Evolution of Structural, Vibrational, Dielectric, and Ferroelectric Properties of Ba1−xSrxTiO3 Ceramics (x = 0, 0.1, 0.2, 0.3, 0.4 and 0.45)
Source: Materials (Basel). 2023 Sep 20;16(18):6316. doi: 10.3390/ma16186316 (PMC10532674; doi:10.3390/ma16186316)
Supplement: Supplementary file 1 [file materials-16-06316-s001.zip › materials-2458718-supplementary.pdf]

Temperature and E-poling evolution of structural, thermal, vibrational, dielectric and ferroelectric properties of $Ba_{1-x}Sr_xTiO_3$ ceramics (x=0, 0.1, 0.2, 0.3, 0.4 and 0.45)

J.Suchanicz[1]*, D.Sitko[2], K.Stanuch[3], K.Świerczek[4], G.Jagło[5*], A.Kruk[5], K.Kluczewska-Chmielarz[5], K.Konieczny[5], P.Czaja[5], J.Aleksandrowicz[6], W.Wieczorek[6], J.Grygierek[6], M.Sokolowski[7], G.Stachowski[8], M.Antonova[9], A.Sternberg[9]

[1] Department of Mechanical Engineering and Agrophysics, University of Agriculture in Krakow, Balicka 120, 31-120, Krakow, Poland

[2] Faculty of Exact & Natural Sciences, Pedagogical University, ul Podchorazych 2, 30-084 Krakow, Poland

[3] Institute of Physics, Pedagogical University, ul. Podchorazych 2, 30-084 Krakow, Poland

[4] Faculty of Energy and Fuels, Department of Hydrogen Energy, AGH-University of Science
& Technology, al.Mickiewicza 30, 30-059 Krakow, Poland

[5] Institute of Technology, Pedagogical University, ul. Podchorazych 2, 30-084 Krakow, Poland

[6] Faculty of Materials Science and Ceramics, AGH-University of Science & Technology, 30-059 Krakow, al.Mickiewicza 30, Poland

[7] Faculty of Computer Science, Electronics and Telecommunications, AGH-University of Science & Technology, 30-059 Krakow, al.Mickiewicza 30, Poland

[8] Astronomical Observatory, Jagiellonian University, Orla 171, 30-244 Krakow, Poland

[9] Institute of Solid State Physics, University of Latvia, Riga, Latvia

* Corresponding author: jan.suchanicz@urk.edu.pl; Grzegorz Jagło: grzegorz.jaglo@up.krakow.pl

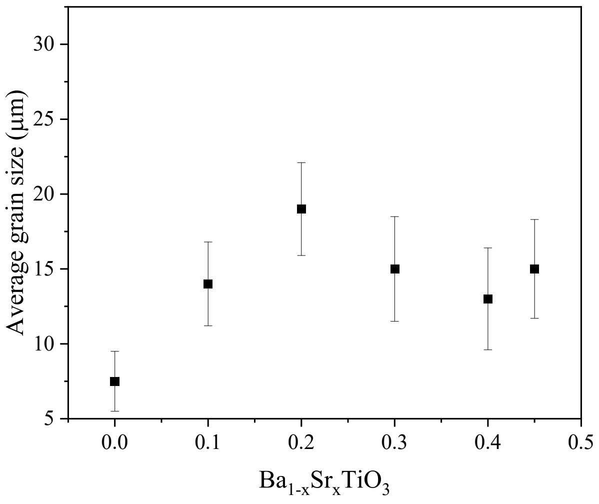

Supplementarny Figure S1. Average grain size in function of Sr content with error bars.

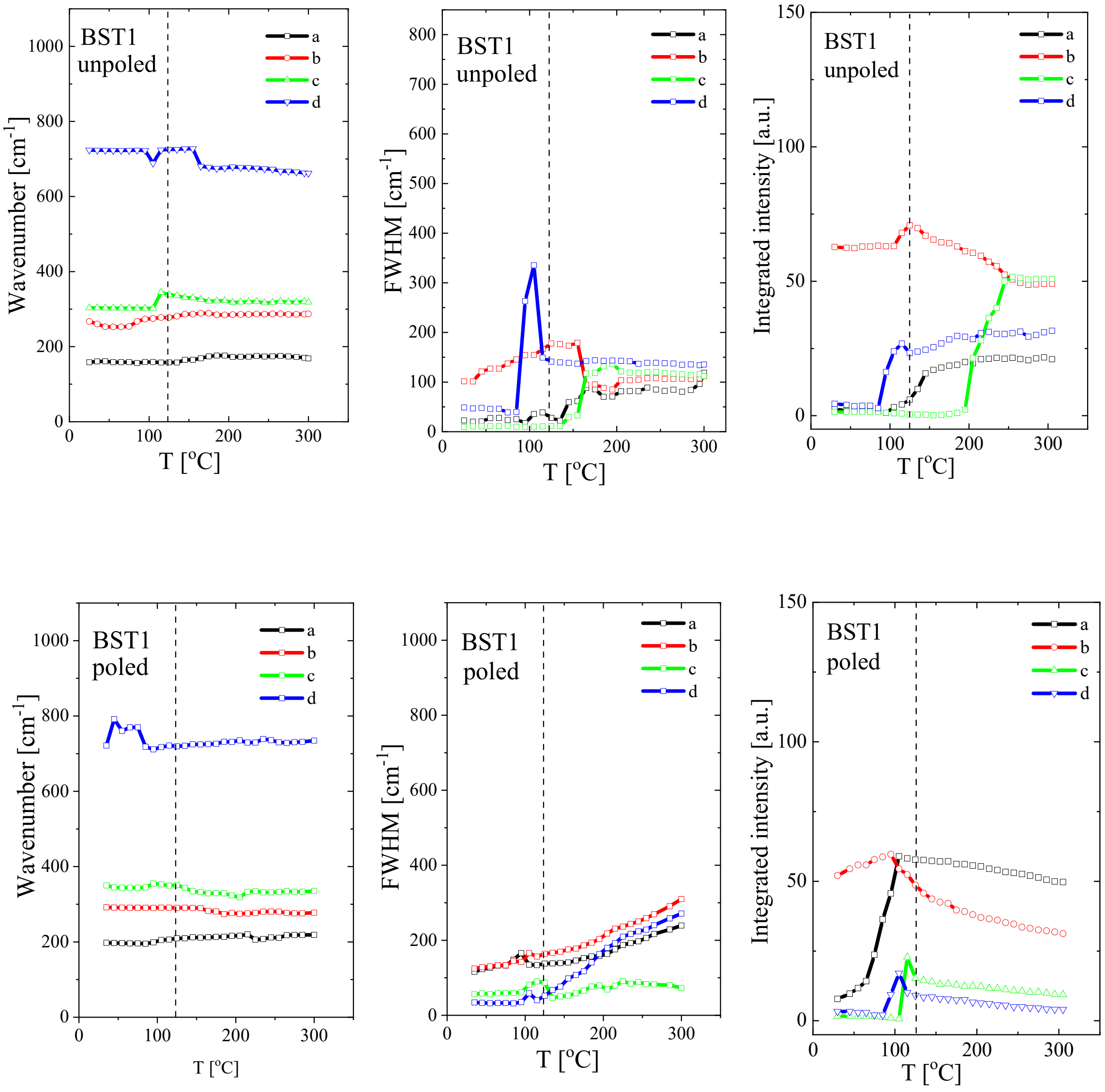
BST1
unpoled
BST1
poled
Wavenumber [cm-1]
FWHM [cm-1]
Integrated intensity [a.u.]
T [°C]
a
b
c
d

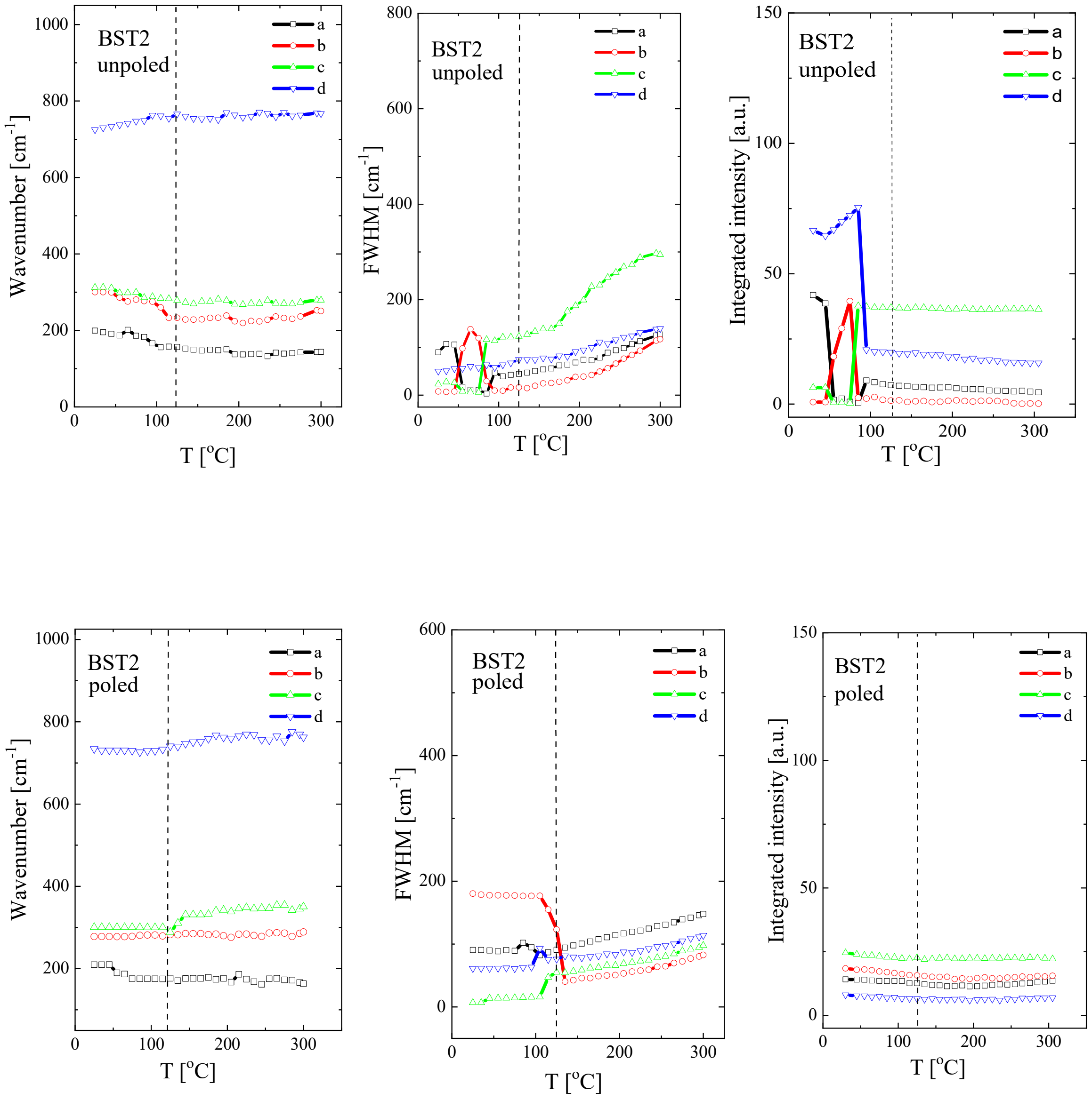

BST2
unpoled
a
b
c
d
Wavenumber [cm-1]
T [oC]
FWHM [cm-1]
Integrated intensity [a.u.]
BST2
poled
0
100
200
300
400
600
800
1000
50
150

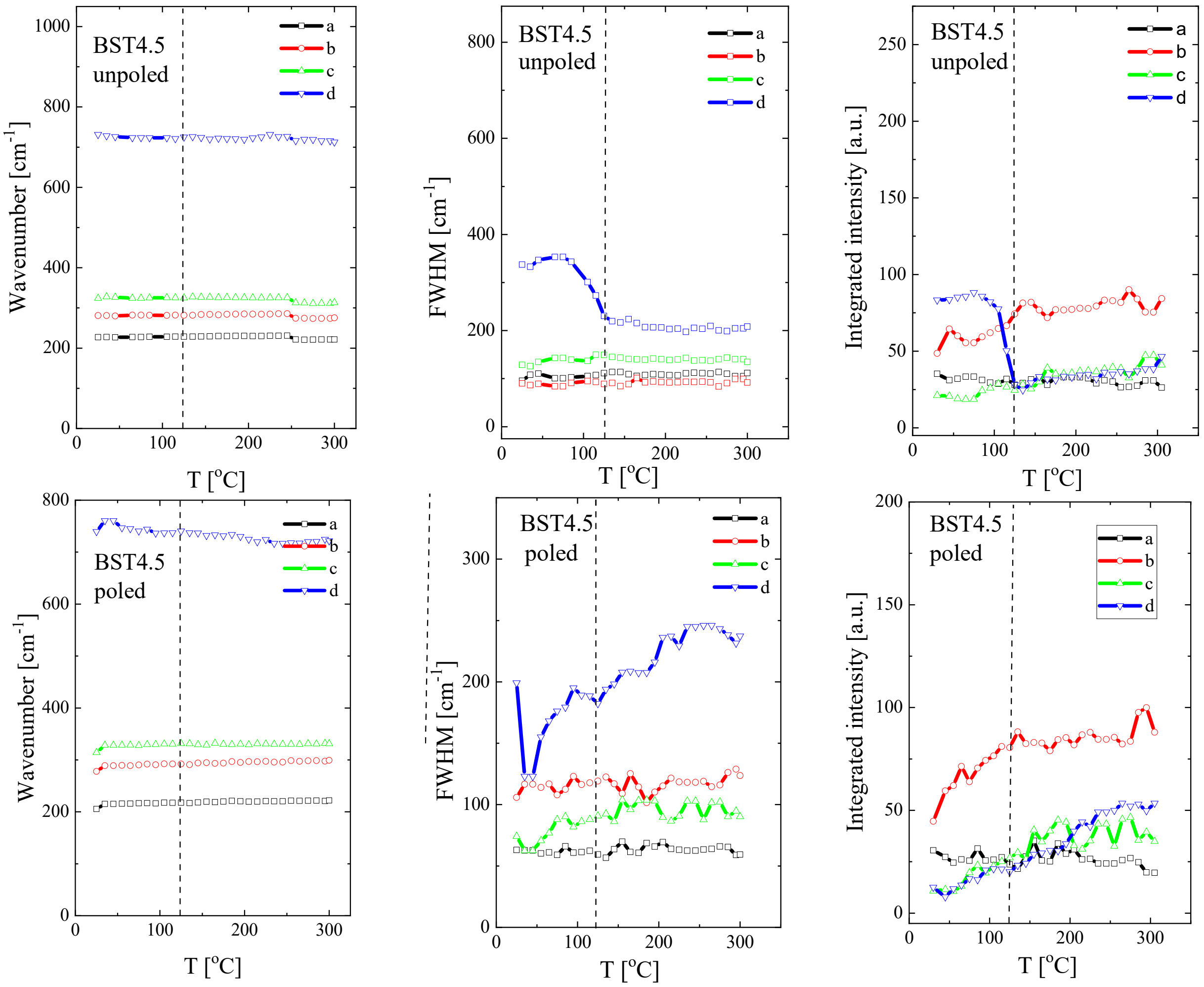

Supplementary Figure S2. Temperature evolution of wavenumbers, FWHM and integrated intensity of BST1, BST2 and BST4.5 ceramics. On each Figures, the points are fitting parameter after deconvolution.
